# Human Placental Mesenchymal Stem Cells Relieve Primary Sclerosing Cholangitis via Upregulation of TGR5 in Mdr2^−/−^ Mice and Human Intrahepatic Cholangiocyte Organoid Models

**DOI:** 10.34133/research.0207

**Published:** 2023-08-17

**Authors:** Qigu Yao, Wenyi Chen, Yingduo Yu, Feiqiong Gao, Jiahang Zhou, Jian Wu, Qiaoling Pan, Jinfeng Yang, Lingling Zhou, Jiong Yu, Hongcui Cao, Lanjuan Li

**Affiliations:** ^1^ State Key Laboratory for the Diagnosis and Treatment of Infectious Diseases, Collaborative Innovation Center for Diagnosis and Treatment of Infectious Diseases, The First Affiliated Hospital, Zhejiang University School of Medicine, 79 Qingchun Rd., Hangzhou 310003, China.; ^2^ National Clinical Research Center for Infectious Diseases, Hangzhou, China.; ^3^ Key Laboratory of Diagnosis and Treatment of Aging and Physic-chemical Injury Diseases of Zhejiang Province, 79 Qingchun Rd., Hangzhou 310003, China.; ^4^ Jinan Microecological Biomedicine Shandong Laboratory, Jinan 250117, China.

## Abstract

Primary sclerosing cholangitis (PSC) is a biliary disease accompanied by chronic inflammation of the liver and biliary stricture. Mesenchymal stem cells (MSCs) are used to treat liver diseases because of their immune regulation and regeneration-promoting functions. This study was performed to explore the therapeutic potential of human placental MSCs (hP-MSCs) in PSC through the Takeda G protein-coupled receptor 5 (TGR5) receptor pathway. Liver tissues were collected from patients with PSC and healthy donors (*n* = 4) for RNA sequencing and intrahepatic cholangiocyte organoid construction. hP-MSCs were injected via the tail vein into Mdr2^−/−^, bile duct ligation (BDL), and 3,5-diethoxycarbonyl-1,4-dihydrocollidine (DDC) mouse models or co-cultured with organoids to confirm their therapeutic effect on biliary cholangitis. Changes in bile acid metabolic profile were analyzed by liquid chromatography/tandem mass spectrometry (LC-MS/MS). Compared with healthy controls, liver tissues and intrahepatic cholangiocyte organoids from PSC patients were characterized by inflammation and cholestasis, and marked downregulation of bile acid receptor TGR5 expression. hP-MSC treatment apparently reduced the inflammation, cholestasis, and fibrosis in Mdr2^−/−^, BDL, and DDC model mice. By activating the phosphatidylinositol 3 kinase/extracellular signal-regulated protein kinase pathway, hP-MSC treatment promoted the proliferation of cholangiocytes, and affected the transcription of downstream nuclear factor κB through regulation of the binding of TGR5 and Pellino3, thereby affecting the cholangiocyte inflammatory phenotype.

## Introduction

Primary sclerosing cholangitis (PSC) is a biliary disease often accompanied by chronic inflammation of the liver and progressive biliary stricture [1]. No genetic or environmental factors have yet been established for this disease, and there are as yet no known triggers for disease manifestation [2]. Takeda G protein-coupled receptor 5 (TGR5, also known as G protein-coupled bile acid receptor 1 [GPBAR1]) is a G protein-coupled receptor that is widely expressed in large and small bile duct epithelial cells in the liver and is activated by stimulation with bile acid. PSC patients show TGR5 sequence variation, which may be one of the susceptibility factors [3–5]. In addition to promoting bile acid secretion, TGR5 can protect the liver from bile acid overload by regulating the permeability of the bile duct epithelium [6]. TGR5 activation also promotes proliferation and inhibits apoptosis of cholangiocytes [7]. Bile-derived cholangiocyte organoids derived from patients with PSC can recapitulate the inflammatory profile of this disease [8]. The relationship between TGR5 and the inflammatory response of cholangiocytes has yet to be elucidated.

No effective pharmacotherapy has yet been developed for treatment of patients with PSC [9]. Liver transplantation remains the only life-saving option, but disease recurrence occurs in approximately 25% of graft recipients [1]. Mesenchymal stem cells (MSCs), characterized by self-renewal and multi-directional differentiation potential, have shown therapeutic potential for treating a number of diseases, including cardiovascular and autoimmune diseases [10,11]. Human placental-derived MSCs (hP-MSCs) are located in the fetal membrane of the full-term placenta, and can be collected both easily and noninvasively. Previous studies showed that hP-MSCs have a strong immunosuppressive ability and promote cell regeneration in patients with COVID-19 [12], acute liver failure [13], and liver cirrhosis [14]. Extracellular vesicles derived from human bone marrow MSCs can target the liver, reduce bile acid and alanine aminotransferase levels, decrease the content of T cells, and ameliorate liver fibrosis [15]. The effectiveness of hP-MSC treatment has not been evaluated in PSC animal models, and there have been insufficient studies of the mechanism by which hP-MSCs regulate cholangiocytes.

In this study, we collected liver tissue from PSC patients, constructed intrahepatic cholangiocyte organoids, and developed a number of cholestasis disease models to clarify the localization and differential expression of TGR5. The differential expression of interleukin (IL)-8 and its homologues C-X-C motif chemokine ligand 1/2 (CXCL1/2), and their effects on the expression of TGR5 were examined in human patients and mouse models. Intrahepatic cholangiocyte organoids and mouse models were used to explore the effectiveness of hP-MSCs for treatment of PSC and the associated changes in bile acid metabolism. Finally, we examined the potential mechanism underlying changes in TGR5 expression and downstream regulation of cholangiocytes in PSC after hP-MSC treatment. This study was performed to explore the pathomechanism of PSC and clarify the feasibility and mechanism of action of hP-MSCs in the treatment of PSC.

## Results

### Characteristics of liver tissue-derived intrahepatic cholangiocyte organoid from PSC patients and healthy donors

**Fig. 1.**
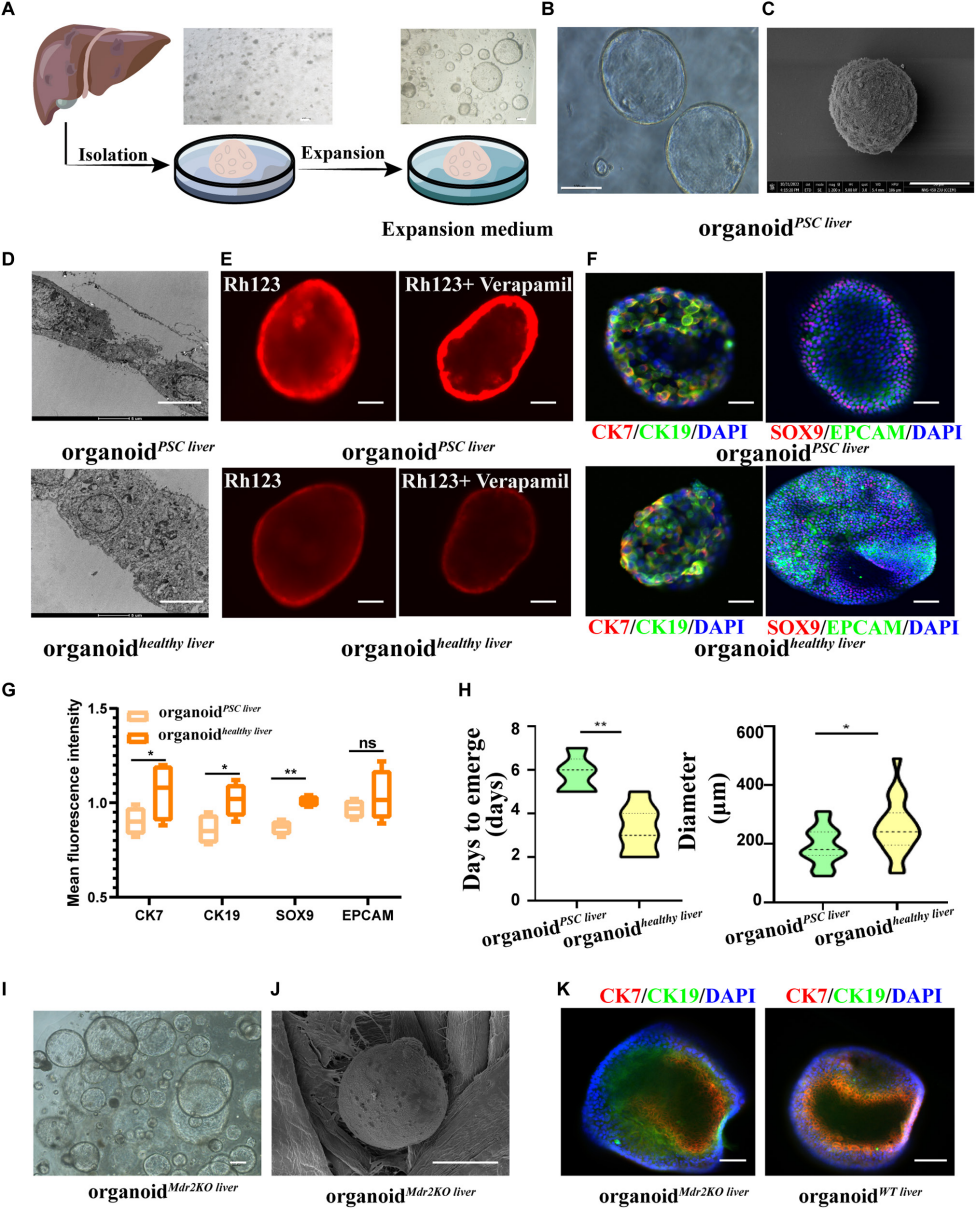
Construction of liver tissue-derived intrahepatic cholangiocyte organoid from PSC patients and healthy controls. (A) Schematic of the experimental strategy for isolation and culture of intrahepatic cholangiocyte organoids. (B) Light micrograph of organoid*^PSC liver^*. Scale bar: 100 μm. (C) Scanning electron microscopy (SEM) micrograph of organoid*^PSC liver^*. Scale bar: 30 μm. (D) Transmission electron microscopy micrographs of organoid*^PSC liver^* and organoid*^healthy liver^*. Scale bar: 5 μm. (E) R123 experiments demonstrated that organoids had barrier function. The fluorescence intensity of organoid^healthy liver^ and organoid^PSC liver^ was compared when Rh123 buffer was added in organoids at 37 °C for 2 h with or without 10 μM verapamil. (F) Organoid*^healthy liver^* and organoid*^PSC liver^* expressed stemness markers (SOX9) and cholangiocyte markers (CK7, CK19, and EPCAM). Scale bar: 100 μm. (G) Quantitative analysis of SOX9, CK7, CK9, and EPCAM fluorescence intensity between organoid*^healthy liver^* and organoid*^PSC liver^*. (H) The appearance time of primary intrahepatic cholangiocyte organoids derived from PSC liver (organoid*^PSC liver^*) and healthy donor liver (organoid*^healthy liver^*), and the diameter of organoid on day 8. (I) Light micrograph of organoid*^Mdr2KO liver^*. Scale bar: 100 μm. (J) SEM micrograph of organoid*^Mdr2KO liver^*. Scale bar: 30 μm. (K) Immunofluorescence analysis indicated positivity for cholangiocyte marker proteins CK7 and CK19 in organoid*^Mdr2KO liver^* and organoid*^WT liver^*. Scale bar: 100 μm. *P < 0.05, **P < 0.01, not significant (ns) > 0.05.

Human liver tissue formed intrahepatic cholangiocyte organoids (named as organoid*^PSC liver^* and organoid*^healthy liver^*) (Fig. 1A). Organoid*^PSC liver^* and organoid*^healthy liver^* showed higher expression levels of the bile duct cell marker CK19 and stemness markers SOX9 when compared with liver tissue, but did not express the liver markers albumin (ALB) and hepatocyte nuclear factor 4 alpha (HNF4A) as determined by quantitative polymerase chain reaction (qPCR; Fig. S1A). Single-layer spherical structure was observed under light microscopy and scanning electron microscopy (SEM) (Fig. 1B and C). Compared with organoid*^healthy liver^*, organoid*^PSC liver^* was more chaotic, with more cells tending to senescence, and bile duct cholangiocyte cilia were reduced as determined by transmission electron microscopy (Fig. 1D). After adding Rhodamine 123 for 2 h with or without Verapamil, organoid*^PSC liver^* showed faster uptake of Rhodamine 123 as determined by laser confocal microscopy, indicating that the barrier function was weakened in organoids derived from tissue of PSC patients (Fig. 1E). Immunofluorescence staining showed that intrahepatic cholangiocyte organoids expressed CK7, CK19, SRY-Box transcription factor 9 (SOX9), and epithelial cell adhesion molecule (EPCAM) (Fig. 1F). Compared with organoid*^PSC liver^*, organoid*^healthy liver^* showed higher levels of expression of the bile duct cell marker CK19 and stemness markers SOX9 but did not express marked difference in the epithelial markers (EPCAM). The same result was also repeated in quantitative analysis of SOX9, CK7, CK19, and EPCAM fluorescence intensity between organoid*^healthy liver^* and organoid*^PSC liver^* (Fig. 1G). Besides, the appearance time of organoid*^PSC liver^* was significantly longer than that for organoid*^healthy liver^* (5.80 ± 0.84 vs. 3.29 ± 1.11 days, respectively, *n* = 4), and the diameter of organoid*^PSC liver^* on day 8 was significantly smaller than that of organoid*^healthy liver^* (191.50 ± 63.43 vs. 256.00 ± 94.45 μm, respectively, *n* = 30) (Fig. 1H).

After digestion, isolation, and cultivation of Mdr2^−/−^ mouse liver tissue for 3 to 5 days, intrahepatic cholangiocyte organoids (organoid*^Mdr2KO liver^*) formed with morphology similar to that of organoid*^PSC liver^* on light microscopy and SEM (Fig. 1I, J). The qPCR analysis showed that organoid*^Mdr2KO liver^* had apparently increased LGR5, SOX9, and CK19 expression, and apparently reduced ALB and HNF4A expression in comparison with Mdr2^−/−^ mouse liver tissue (Fig. S1B). Immunofluorescence analyses showed that organoid*^WT liver^* expressed stronger fluorescence in CK7 and CK19, compared with organoid*^Mdr2KO liver^* (Fig. 1K). The same result was also repeated in the quantitative analysis of CK7 and CK19 fluorescence intensity between organoid*^healthy liver^* and organoid*^PSC liver^* (Fig. S2A). The appearance time of organoid*^Mdr2KO liver^* was significantly longer than that for organoid*^WT liver^* (5.00 ± 0.76 vs. 3.25 ± 1.04 days, respectively, *n* = 8), and the diameter of organoid*^Mdr2KO liver^* on day 8 was significantly smaller than that of organoid*^WT liver^* (208.57 ± 55.36 vs. 253.57 ± 59.46 μm, respectively, *n* = 30) (Fig. S2B).

### Transcriptome analysis revealed the bile acid metabolic profile and inflammation in PSC patients and the organoid*^PSC liver^* model

Table S1 summarizes the clinical characteristics of 4 PSC patients. Hematoxylin and eosin (HE) staining and Sirius Red staining revealed robust liver damage and collagen fibers (Fig. S3). RNA sequencing analysis revealed the differences in gene expression between liver and organoid specimens from the PSC patient and healthy donor groups. Data from 24 cases were examined using StringTie software for the sequences of known genes, and FPKM was used to calculate the measurement index for the expression of known genes (Fig. S4A). Differentially expressed genes (DEGs) were defined by *P* < 0.05 and fold change > 1.

**Fig. 2.**
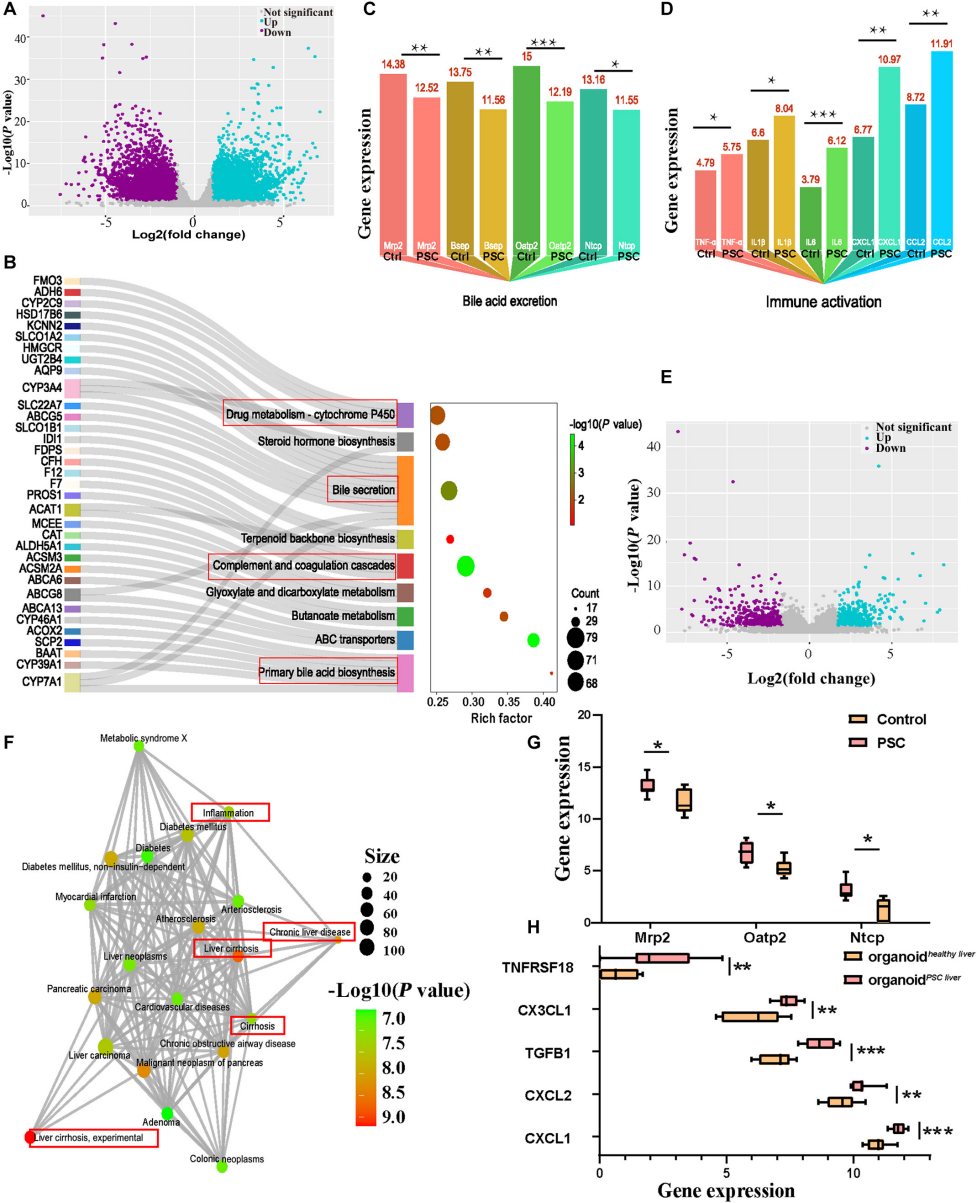
Transcriptome analysis revealed the bile acid metabolic profile and inflammation in PSC patients and organoid*^PSC liver^*. (A) Volcano plot of DEGs. Blue dots indicate upregulated genes and purple dots indicate downregulated genes in PSC patients. (B) Sankey dot pathway enrichment of DEGs. Dot size represents the number of DEGs, and the dot color represents the corresponding *P* value. Red boxes represent pathways that may explain the more disordered bile acid metabolism and inflammation in PSC patients than healthy controls. (C) Histogram of bile acid excretion-related gene expression. (D) Histogram of immune activation-related gene expression. (E) Volcano plot of DEGs between organoid*^PSC liver^* and organoid*^healthy liver^*. Organoid (passages 3 and 7) were cultured for 96 h and then were used to harvest RNA for RNA-sequencing (RNA-seq). Blue dots indicate upregulated genes and purple dots indicate downregulated genes in organoid*^PSC liver^*. (F) Disease enrichment analysis of sets of DEGs. (G) Box plot of bile acid excretion-related gene expression in organoid*^PSC liver^* and organoid*^healthy liver^* (*n* = 8 for each group). (H) Box plot of immune activation-related gene expression in organoid*^PSC liver^* and organoid*^healthy liver^* (*n* = 8 for each group). *P < 0.05, **P < 0.01, ***P < 0.001.

A total of 9,973 DEGs were identified between the liver tissues of PSC patients and healthy controls (Fig. 2A). Hierarchical clustering was performed to determine the overall differences between PSC and healthy control liver tissue (Fig. S4B). With the functional annotation and classification of disease types in the DisGeNET disease database, DEGs were enriched in liver cirrhosis (Fig. S5A). Kyoto Encyclopedia of Genes and Genomes (KEGG) and Gene Ontology (GO) analyses showed that DEGs were enriched in bile acid metabolism and immune activation (Fig. 2B and Fig. S5B). The results of RNA-Seq showed that bile secretion-related genes (Mrp2, Bsep, Oatp2, and Ntcp) were significantly downregulated (Fig. 2C), while immune activation-related genes (tumor necrosis factor-α [TNF-α], IL-1β, IL-6, CXCL1, and CCL2) were significantly elevated in PSC liver tissue compared with healthy control liver tissue (Fig. 2D).

A total of 1,341 DEGs were identified in organoid; 637 were upregulated and 704 were downregulated (Fig. 2E). Hierarchical clustering was performed to determine the overall differences between organoid*^PSC liver^* and organoid*^healthy liver^* (passages 3 and 7, *n* = 16) (Fig. S6A). There were 368 and 364 DEGs between organoid*^PSC liver^* and organoid*^healthy liver^* at passages 3 and 7, respectively, indicating that organoids still maintained original liver characteristics after passage (Fig. S6B and C). Therefore, passage 3 to 7 organoids were used in subsequent experiments. DEGs were also mapped to liver fibrosis and inflammatory diseases (Fig. 2F). The differences in intrahepatic cholangiocyte organoids were similar to those of liver tissues; i.e., bile secretion-related genes (Mrp2, Oatp2, and Ntcp) were significantly downregulated (Fig. 2G), and immune activation-related genes (TNFRSF18, CX3CL1, TGF-β1, CXCL2, and CXCL1) were significantly upregulated (Fig. 2H). Besides, we also found that PSC derived-organoids tended to secrete a series of cholangiocyte senescence (P16 and P21), and profibrotic cytokines (CTGF and PDGF) (Fig. S7A to D). However, no difference was found in angiogenesis ability between organoid*^PSC liver^* and organoid*^healty control^* by RNA-Seq analysis (Fig. S7E).

Taken together, the results of transcriptome analyses showed that the disease annotations of DEGs from PSC were enriched in liver fibrosis and inflammation. The DEGs were enriched in bile acid metabolism and immune activation-related pathways, similar to the results in PSC intrahepatic cholangiocyte organoids.

### hP-MSC treatment alleviated liver fibrosis and inflammation in mouse models of sclerosing cholangitis

**Fig. 3.**
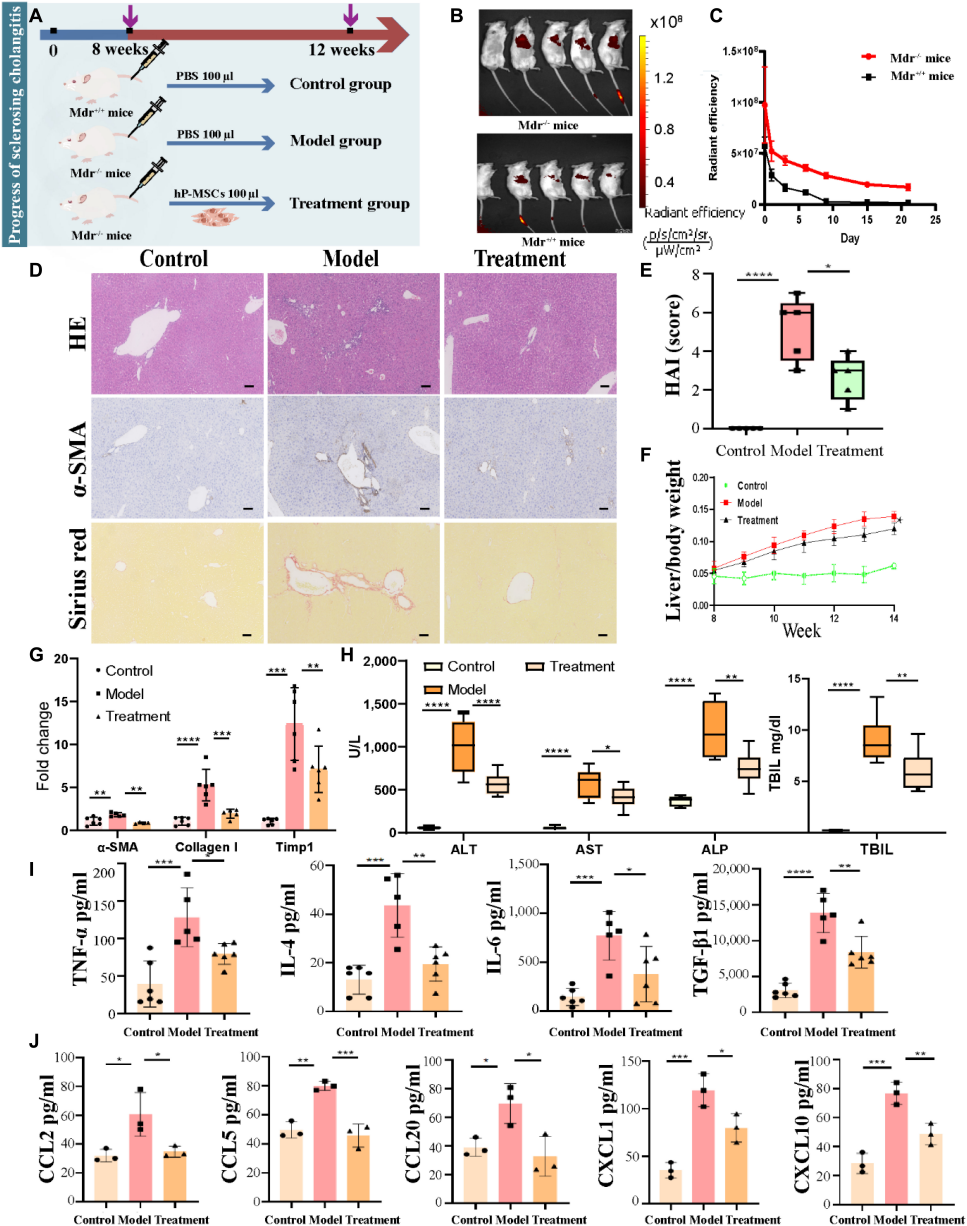
Efficacy of hP-MSC treatment in the Mdr2^−/−^ mouse model of sclerosing cholangitis. (A) Schematic representation of the methodology used for treatment of a sclerosing cholangitis mouse model with hP-MSCs. Mdr2^+/+^ mice were used as the control group (*n* = 30), Mdr2^−/−^ mice were used as the model group (*n* = 30), and hP-MSC-treated Mdr2^−/−^ mice were used as the treatment group (*n* = 30). (B) Whole-body fluorescence imaging of Mdr2^−/−^ mice and Mdr2^+/+^ mice treated with hP-MSCs. Images were taken 6 h after tail vein injection. (C) Quantitative analysis of fluorescence intensity of DiR-labeled hP-MSCs in Mdr2^−/−^ mice and Mdr2^+/+^ mice at various time points (6 h, 24 h, day 3, day 6, day 9, day 15, and day 21). (D) Analysis of hematoxylin and eosin (HE)-stained sections showed that hP-MSC treatment improved the inflammatory infiltration and cell necrosis in Mdr2^−/−^ mice. Immunohistochemical analysis of α-smooth muscle actin (α-SMA) and Sirius red staining showed that MSC treatment ameliorated liver fibrosis and collagen proliferation in mice. Scale bar: 100 μm. (E) Evaluation of therapeutic efficacy in Mdr2^−/−^ mice by histological activity index (HAI) score (*n* = 5 for each group). (F) Liver/body weight percentage in Mdr2^−/−^ mice (*n* = 8 for each group). (G) Fibrosis-related gene (a-SMA, Collagen I, and Timp 1) mRNA levels in whole liver tissue among 3 groups (*n* = 5 to 6 for each group). (H) Serum levels of ALT, AST, ALP, and total bilirubin (TBIL) (*n* = 8 for each group). (I and J) Serum levels of inflammatory factors (TNF-α, IL-4, IL-6, and TGF-β1) and chemokines (CCL2, CCL5, CCL20, CXCL1, and CXCL10) in the Mdr2^−/−^ mice (*n* = 3 to 6 for each group). *P < 0.05, **P < 0.01, ***P < 0.001, ****P < 0.0001.

The results of phenotypic identification and multilineage differentiation of hP-MSCs are shown in Figs. S8 and S9. hP-MSC treatment of sclerosing cholangitis mouse models (bile duct ligation [BDL] and Mdr2^−/−^) is shown in Fig. 3A and Fig. S10A, respectively. The MSC homing capacity toward inflammation and injured sites is an essential process to a successful cell therapy. Mdr2^−/−^ mice and Mdr2^+/+^ mice were injected intravenously with hP-MSCs labeled with the fluorescent dye 1,1′-dioctadecyl-3,3,3′,3′-tetramethylindotricarbo-cyanine-iodide (DiR) for long-term follow-up. Fluorescence of DiR-labeled hP-MSCs could be detected at 6 h and 1, 4, 7, 9, 15, and 21 days after injection, and decreased gradually thereafter (Fig. 3B and C). In general, the fluorescence intensity was stronger in Mdr2^−/−^ mice (approximately 150% to 200%), compared with Mdr2^+/+^ mice every detected time. DiR-labeled cells were mainly distributed in the lungs and liver, while no labeled cells were found in the heart, spleen, and intestine (Fig. S11). In addition, hP-MSCs were observed to differentiate into hepatocytes at week 4 in Mdr2^−/−^ mice (Fig. S12). After hP-MSC administration via the tail vein in the 3 mouse models, immune infiltration and necrosis of liver tissue were apparently reduced, while the fibrosis level was apparently decreased in the hP-MSC treatment group (Fig. 3D and G and Fig. S10B). Histological activity index (HAI) score and liver/body weight ratio were also decreased in the hP-MSC treatment group (Fig. 3E and F and Fig. S10C). hP-MSCs significantly reduced mortality in BDL models with greater alleviation of disease progression (Fig. S10D). Serum biochemical indexes, such as ALT, AST, ALP, and TBIL, were significantly decreased (Fig. 3H and Fig. S10E), and TNF-α, IL-4, IL-6, TGF-β1, CCL2, CCL5, CCL20, CXCL1, and CXCL10 were also significantly decreased in the hP-MSC treatment group (Fig. 3I and J), but there was no significant differences in IL-2, CXCL9, and CCL11 (Fig. S13) on cytokine multiplex assays (LEGENDplex Multi-cytokine ELISA kits; BioLegend, San Diego, CA, USA).

**Fig. 4.**
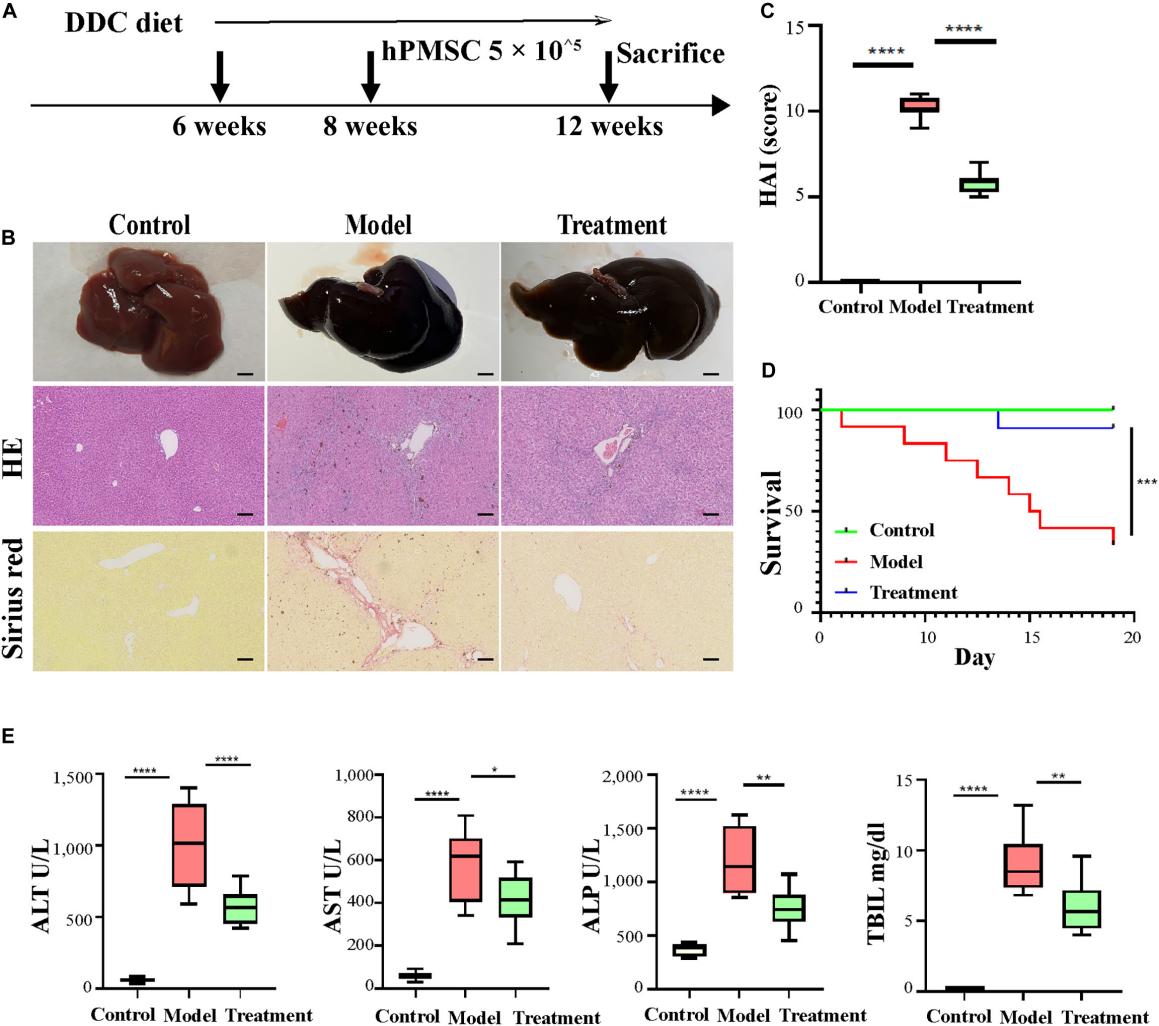
Efficacy of hP-MSC treatment in the DDC mouse model of sclerosing cholangitis. (A) Schematic representation of the methodology used for treatment of DDC mice with hP-MSCs. C57Bl/6 mice were used as the control group (*n* = 30), 0.1% 3,5-diethoxycarbonyl-1,4-dihydrocollidine (DDC)-fed mice were used as the model group (*n* = 30), and hP-MSC-treated DDC mice were used as the treatment group (*n* = 30). (B) HE and Sirius red staining of sections of the liver and gallbladder from Control, Model, and Treatment groups. Scale bar: 100 μm. (C) Evaluation of therapeutic efficacy in DDC mice by HAI score (*n* = 5 for each group). (D) Survival rates in the Control, Model, and Treatment after 30 days (*n* = 8 for each group). (E) Serum levels of ALT, AST, ALP, and TBIL (*n* = 8 for each group). *P < 0.05, **P < 0.01, ***P < 0.001, ****P < 0.0001.

Treatment with hP-MSCs apparently ameliorated hepatic fibrosis and inflammation in mouse models of sclerosing cholangitis. hP-MSC treatment of 3,5-diethoxycarbonyl-1,4-dihydrocollidine (DDC) mouse models is shown in Fig. 4A. After hP-MSC administration via the tail vein in the mouse models, immune infiltration and necrosis of liver tissue were significantly reduced, while the fibrosis level was significantly decreased in the hP-MSC treatment group **(**Fig. 4B). HAI score was also decreased in the hP-MSC treatment group (Fig. 4C). hP-MSCs significantly reduced mortality in DDC models with greater alleviation of disease progression (Fig. 4D). Serum biochemical indexes, such as ALT, AST, ALP, and TBIL, were significantly decreased (Fig. 4E).

### Treatment with hP-MSCs ameliorated alterations in bile acid metabolism in Mdr2^−/−^ mice by activation of TGR5 rather than FXR in cholangiocytes

**Fig. 5.**
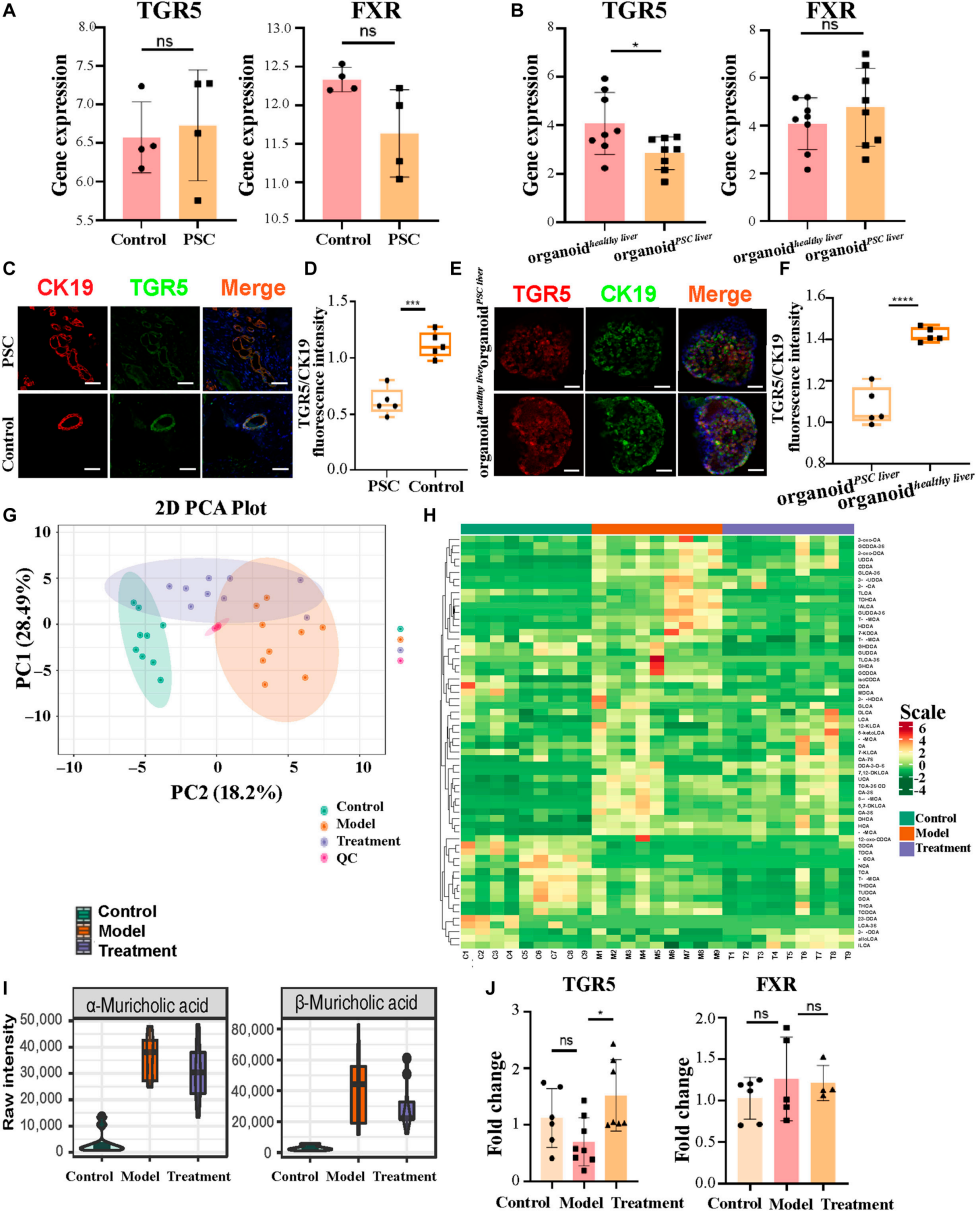
hP-MSC treatment ameliorated the changes in bile acid metabolism in the Mdr2^−/−^ mouse model of sclerosing cholangitis. (A) TGR5 mRNA levels in whole liver tissue from PSC patients and healthy controls (*n* = 4 for each group). (B) TGR5 and FXR mRNA expression levels in organoid*^PSC liver^* and organoid*^healthy liver^* (*n* = 8 for each group). (C) Fluorescence staining of TGR5 and CK 19 in cholangiocytes of PSC patients and healthy controls. Scale bar: 100 μm. (D) Fluorescence staining intensity of TGR5/CK19 in cholangiocytes of PSC patients and healthy controls (*n* = 5 for each group). (E) Fluorescence staining of TGR5 in organoid*^PSC liver^* and organoid*^healthy liver^*. Scale bar: 100 μm. (F) Fluorescence staining intensity of TGR5/CK19 in organoid*^PSC liver^* and organoid*^healthy liver^* (*n* = 5 for each group). (G) 2D PCA of bile acids in the control, model, and treatment group (*n* = 9 for each group). (H) Heat map of hierarchical clustering of bile acids among 3 groups: Mdr2^+/+^ mice (control group, green dots), Mdr2^−/−^ mice (model group, orange dots), and hP-MSC-treated Mdr2^−/−^ mice (treatment group, purple dots). (I) The levels of α-muricholic acid and β-muricholic acid in Mdr2^−/−^ mice decreased after hP-MSC treatment (*n* = 9 for each group). (J) mRNA levels of TGR5 and FXR in Mdr2^+/+^ mice (control group), Mdr2^−/−^ mice (model group), and hP-MSC-treated Mdr2^−/−^ mice (treatment group) were determined by qPCR. (*n* = 4 to 7 for each group). *P < 0.05, **P < 0.01, ***P < 0.001, ****P < 0.0001, not significant (ns) > 0.05.

RNA-Seq analysis showed that there were no differences in bile acid receptor TGR5 and farnesoid X receptor (FXR) mRNA expression between PSC and healthy liver tissues (Fig. 5A). However, the levels of TGR5 gene expression in organoid*^PSC liver^* were significantly lower than those in organoid*^healthy liver^* (Fig. 5B). Compared with healthy donor tissue, the fluorescence staining intensity and immunohistochemical color depth of TGR5 in cholangiocytes of PSC patients were decreased (Fig. 5C and D and Fig. S14A), and the fluorescence of TGR5 in the organoid*^PSC liver^* was also reduced (Fig. 5E and F), while the level of FXR protein was not significantly different in liver tissues or organoids between PSC and healthy control. As TGR5 is also highly expressed in hepatocytes and macrophages of PSC liver (Fig. S14B), TGR5 expression may be specifically decreased in cholangiocytes of PSC patients.

The liver tissues of Mdr2^+/+^ mice (control group), Mdr2^−/−^ mice (model group), and hP-MSC-treated Mdr2^−/−^ mice (treatment group) were analyzed by liquid chromatography/mass spectrometry. On 2-dimensional (2D) principal component analysis (PCA), the 3 groups could be well distinguished and clustered, and the characteristics of the hP-MSC treatment group were intermediate between those of the control group and model group (Fig. 5G). The heat map of bile acid hierarchical clustering among the 3 groups showed that bile acid deposition was higher in the model group than the control group, and was ameliorated in the treatment group (Fig. 5H); in particular, α-muricholic acid and β-muricholic acid were significantly decreased in the treatment group (Fig. 5I). In KEGG analysis, DEGs were enriched in primary bile acid synthesis and bile secretion (Fig. S15). qPCR of mouse liver tissue showed that hP-MSCs significantly improved bile acid metabolism (TGR5 mRNA level was significantly increased), reduced bile acid synthesis (levels of CYP7A1 and CYP27B1 mRNA were significantly decreased), and increased bile acid secretion (MRP2 mRNA level was significantly increased). However, there were no differences in FXR mRNA level expression (Fig. 5J and Fig. S16). Compared with Mdr2^+/+^ mice, senescence-related P16 and P21 protein expression were specifically decreased in Mdr2^−/−^ mice, and could be rescued by hP-MSC treatment in the Mdr2^−/−^ model (Fig. S17A and B). TGR5 protein expression may be specifically decreased in cholangiocytes of Mdr2^−/−^ mice, and a significant increase was also observed in the Mdr2^−/−^ model after hP-MSC treatment (Fig. S17C), while the level of FXR protein was not significantly different in liver tissues or organoids among 3 groups (Fig. S17D).

The changes in CYP7A1, CYP27B1, and MRP2 mRNA levels in liver tissues of the DDC and BDL models after hP-MSC treatment were similar to those seen in the Mdr2^−/−^ model (data not shown). Interestingly, a significant increase in TGR5 mRNA level was also observed in the DDC model after hP-MSC treatment, but not in the more severe cholestasis BDL model. This may have been because a large number of cholangiocytes stimulated the expression of TGR5, leading to a compensatory increase in TGR5 (Fig. S18). Therefore, hP-MSCs improved the bile acid metabolism of Mdr2^−/−^ mice, which may play a role by activating the bile acid receptor TGR5 rather than FXR on cholangiocytes.

### hP-MSC treatment improved the expression of TGR5 in organoid*^Mdr2KO liver^* models by downregulating CXCL1/2

**Fig. 6.**
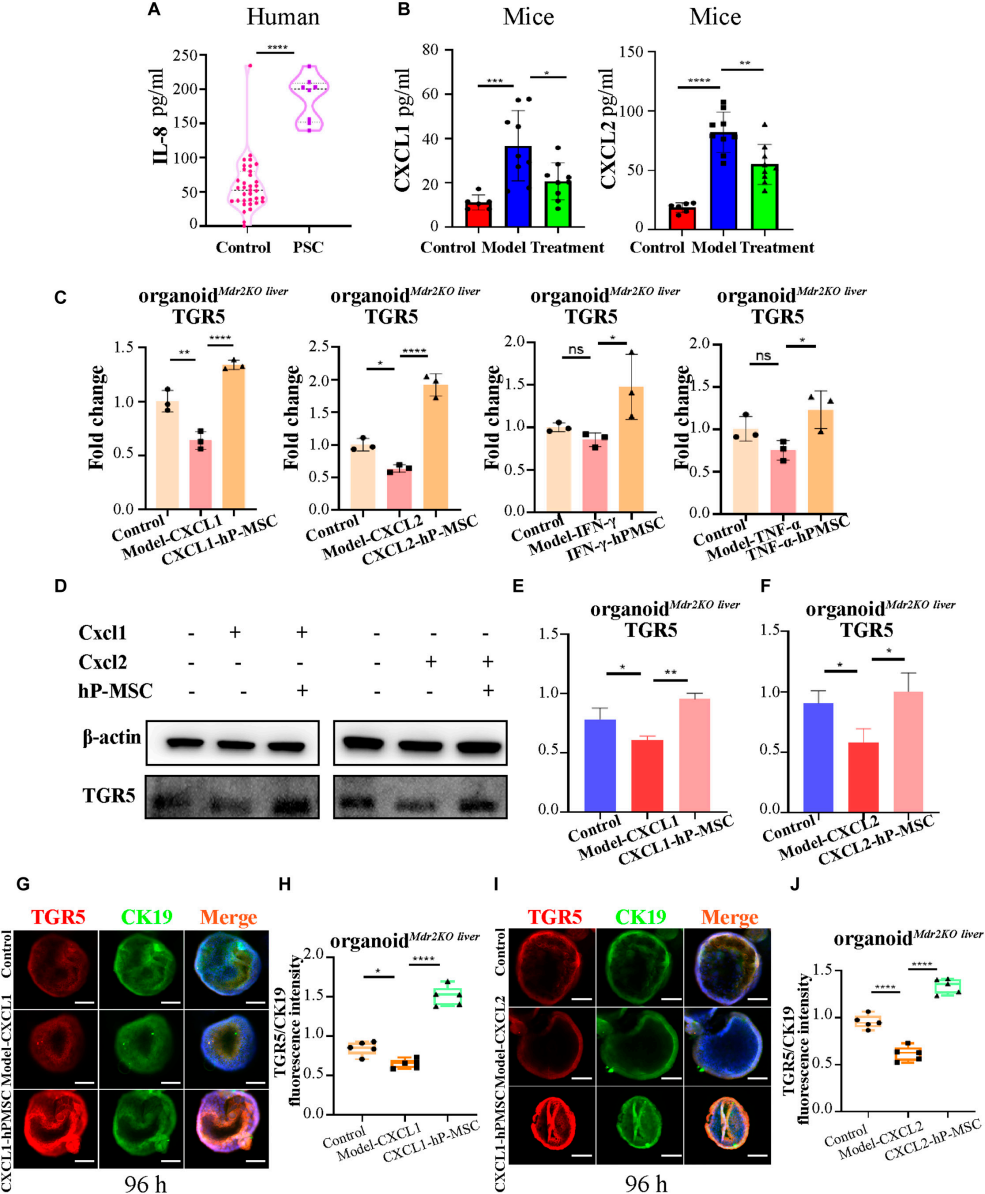
hP-MSC treatment downregulated IL-8 (CXCL1/2), leading to improvement of TGR5 expression in organoid*^Mdr2KO liver^* and alleviation of the pathological process of sclerosing cholangitis. (A) IL-8 levels in PSC patients (*n* = 8) and healthy controls (*n* = 35). (B) CXCL1/2 levels in Mdr2^+/+^ mice (control group), Mdr2^−/−^ mice (model group), and hP-MSC-treated Mdr2^−/−^ mice (treatment group) (*n* = 6 to 9 for each group). (C) TGR5 mRNA levels were decreased in organoid*^Mdr2KO liver^* following stimulation with CXCL1/2 (200 ng/ml) 24 h but not in TNF-α (50 ng/ml) and IFN-γ (50 ng/ml). The effect was ameliorated by co-culture with hP-MSCs for 24 h (*n* = 3 for each group). (D to F) TGR5 protein level was decreased in organoid*^Mdr2KO liver^* following stimulation with CXCL1/2 (200 ng/ml), and the effect was ameliorated by co-culture with hP-MSCs for 24 h by WB analysis (*n* = 3 for each group). (G and I) After stimulation with CXCL1/2 and co-culture with hP-MSCs, organoid*^Mdr2KO liver^* was double-labeled for TGR5 and CK19. Scale bar: 100 μm. (H and J) Quantitative analysis of fluorescence staining intensity of TGR5/CK19 in organoid*^Mdr2KO liver^* following stimulation with CXCL1/2 (200 ng/ml), and the effect was ameliorated by co-culture with hP-MSCs for 24 h (*n* = 5 for each group). *P < 0.05, **P < 0.01, ***P < 0.001, ****P < 0.0001, not significant (ns) > 0.05.

The IL-8 level was significantly increased in the serum of PSC patients (Fig. 6A), similar to its homologues CXCL1/2 in the serum of Mdr2^−/−^ mice. hP-MSC treatment significantly reduced CXCL1/2 levels in the serum of Mdr2^−/−^ mice (Fig. 6B). To examine the relationships between CXCL1/2 and TGR5, we added CXCL1, CXCL2, TNF-α, and IFN-γ separately to organoid*^Mdr2KO liver^* culture medium for 24 h, followed by co-culture with hP-MSCs for a further 24 h. qPCR analysis showed that addition of CXCL1 and CXCL2 led to reductions in the level of TGR5 mRNA in the 2 groups of organoids. There was no difference in TGR5 mRNA level in both the IFN-γ and TNF-α groups (Fig. 6C). Western blotting and immunofluorescence analyses were performed to determine the changes in TGR5 protein level of organoids in the co-culture experiment (Fig. 6D to J), and the results were consistent with the mRNA analyses. These observations showed that the mouse homologues of IL-8, CXCL1/2, could downregulate the expression of TGR5 in organoid*^Mdr2KO liver^* while hP-MSCs had a rescuing effect.

**Fig. 7.**
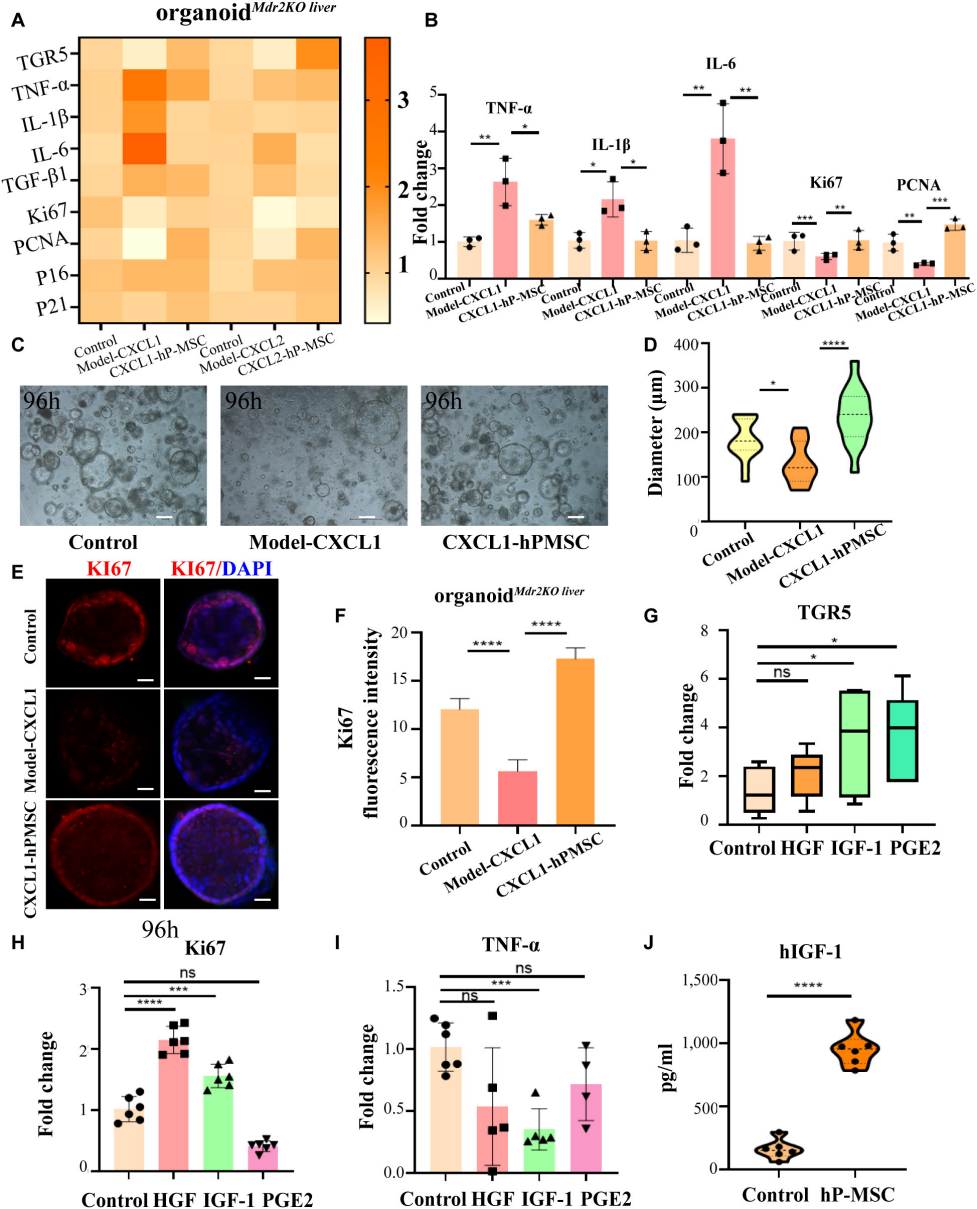
hP-MSC treatment improved the expression of TGR5 in organoid*^Mdr2KO liver^* by downregulating IL-8 (CXCL1/2) and alleviated the pathological process of sclerosing cholangitis. (A) Heat map showing differences in expression of genes related to TGR5, inflammation (TNF-α, IL-1β, IL-6, and TGF-β1), proliferation (Ki-67 and PCNA), and senescence (p16 and p21) between Control, Model-CXCL1, and hP-MSC-CXCL1 groups in organoid*^Mdr2KO liver^* (*n* = 3 for each group). (B) Histogram showing that co-culture with hP-MSCs significantly ameliorated inflammation and proliferation (*n* = 3 for each group). (C) Comparison of Control, Model-CXCL1, and hP-MSC-CXCL1 groups in organoid*^Mdr2KO liver^* at 96 h. Scale bar: 100 μm. (D) The diameters of Control, Model-CXCL1, and hP-MSC-CXCL1 groups in organoid*^Mdr2KO liver^* at 96 h. (E) Immunofluorescence analysis of Ki-67 in organoid*^Mdr2KO liver^* stimulated with CXCL1 and then co-cultured with hP-MSCs. Scale bar: 100 μm. (F) Fluorescence staining intensity of Ki-67 between Control, Model, and CXCL1-hP-MSC in organoid*^Mdr2KO liver^* (*n* = 3 for each group). (G) TGR5 mRNA expression in organoid*^Mdr2KO liver^* after stimulation with HGF (50 ng/ml), IGF-1 (50 ng/ml), and PGE2 (50 ng/ml) 24 h (*n* = 6 for each group). (H) Ki-67 mRNA expression in organoid*^Mdr2KO liver^* after stimulation with HGF (50 ng/ml), IGF-1 (50 ng/ml), and PGE2 (50 ng/ml) 24 h (*n* = 6 for each group). (I) TNF-α mRNA expression in organoid*^Mdr2KO liver^* after stimulation with HGF (50 ng/ml), IGF-1 (50 ng/ml), and PGE2 (50 ng/ml) 24 h (*n* = 6 for each group). (J) IGF-1 protein levels in hP-MSC culture supernatant (24 h) and human MSC medium determined by ELISA (*n* = 6 for each group). *P < 0.05, **P < 0.01, ***P < 0.001, ****P < 0.0001, not significant (ns) > 0.05.

To identify TGR5-dependent downstream phenotypic signals, the expression of the inflammation-related genes (TNF-α, IL-1β, IL-6, and TGF-β1) and proliferation-related genes (Ki-67 and proliferating cell nuclear antigen [PCNA]) were examined. A heat map of the above genes is shown in Fig. 7A. Compared with the organoid*^Mdr2KO liver^*-CXCL1 group, TNF-α, IL-1β, and IL-6 levels were significantly decreased in the hP-MSC-organoid*^Mdr2KO liver^*-CXCL1 group, while Ki-67 and PCNA expression were significantly increased (Fig. 7B). There was no significant difference in TGF-β1, p16, and p21 expression between the 2 groups (Fig. S19A). Compared with the organoid*^Mdr2KO liver^*-CXCL2 group, IL-6 levels were significantly decreased in the hP-MSC-organoid*^Mdr2KO liver^*-CXCL2 group, while Ki-67 and PCNA expression were significantly increased. There was no significant difference in TNF-α, IL-1β, TGF-β1, p16, and p21 expression between the 2 groups (Fig. S19B). Typical organoids of the control, organoid*^Mdr2KO liver^*-CXCL1, and hP-MSC-organoid*^Mdr2KO liver^*-CXCL1 groups are shown in Fig. 7C and D. Immunofluorescence staining for Ki-67 showed that CXCL1 stimulation significantly reduced the proliferation of organoid*^Mdr2KO liver^*, and hP-MSC co-culture significantly ameliorated this effect (Fig. 7E and F).

To examine the mechanisms by which hP-MSCs improved TGR5 expression, proliferation, and the inflammatory phenotype in organoid*^Mdr2KO liver^*, we stimulated organoid*^Mdr2KO liver^* with the 3 cytokines secreted at the highest levels by MSCs, i.e., hepatocyte growth factor (HGF), insulin-like growth factor 1 (IGF-1), and prostaglandin E2 (PGE2). After 96 h of IGF-1 and PGE2 stimulation, TGR5 mRNA expression was significantly increased in organoid*^Mdr2KO liver^* (Fig. 7G). Only IGF-1 stimulation significantly reduced TNF-α expression and significantly increased Ki-67 expression (Fig. 7H and I). At the same time, hP-MSCs were shown to secrete higher concentrations of IGF-1 (Fig. 7J).

### hP-MSC treatment ameliorated the pathological process of mouse cholangiocytes through TGR5/PI3K/ERK and TGR5/Pellino3/NF-κB pathways

**Fig. 8.**
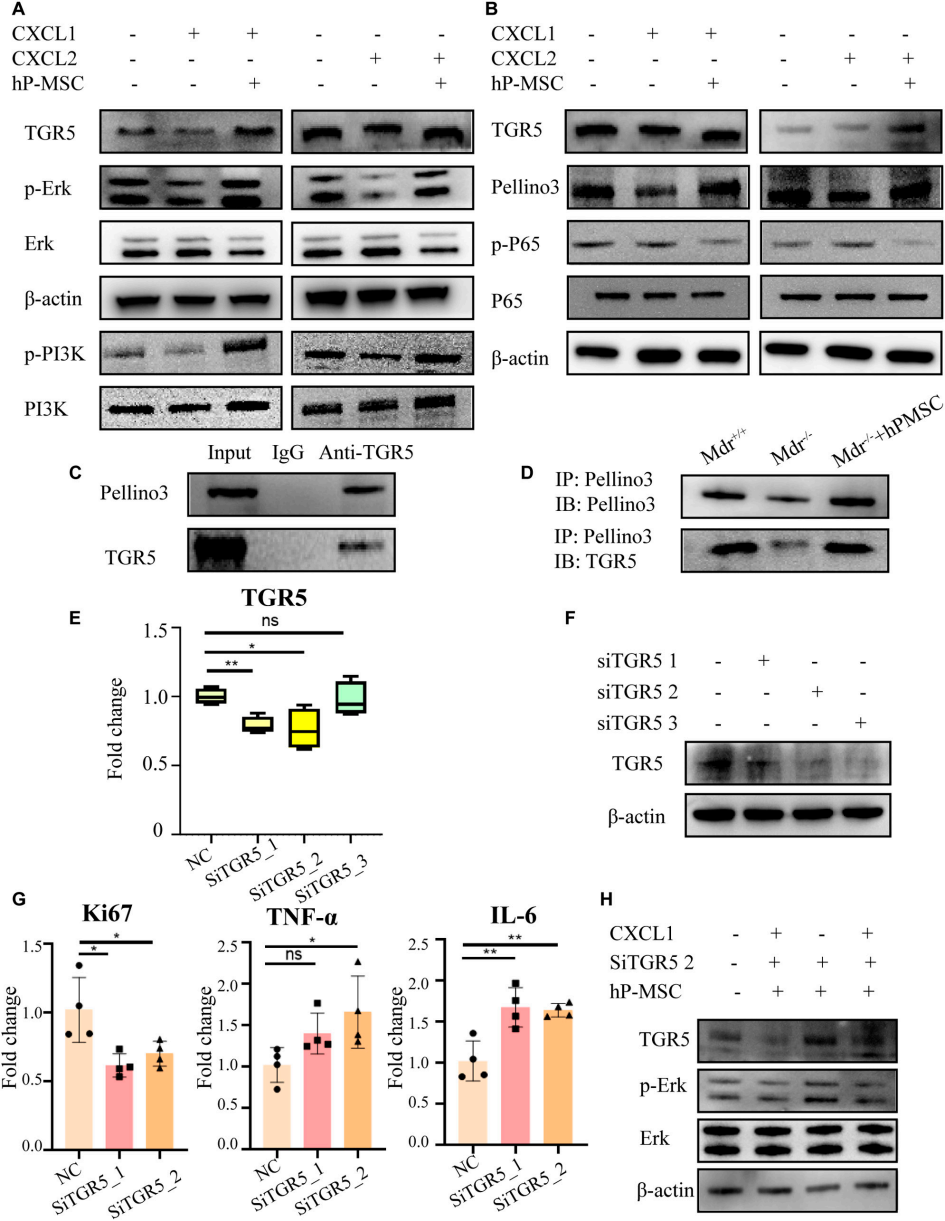
hP-MSC treatment ameliorated the pathological process of sclerosing cholangitis in a mouse model via TGR5/PI3K/ERK and TGR5/Pellino3/NF-κB signaling. (A) Western blotting of TGR5, p-ERK, ERK, β-actin, p-PI3K, and PI3K in organoid*^Mdr2KO liver^* in the CXCL1/2 group treated with hP-MSCs. (B) Western blotting of TGR5, Pellino3, p-p65, p65, and β-actin in organoid*^Mdr2KO liver^* in the CXCL1/2 group treated with hP-MSCs. (C) Western blotting of TGR5 and Pellino3 after co-immunoprecipitation with anti-TGR5 from Mdr2^−/−^ liver tissue. (D) Western blotting after co-immunoprecipitation with anti-TGR5 from liver tissue from Mdr2^+/+^ mice, Mdr2^−/−^ mice, and Mdr2^−/−^ mice treated with hP-MSCs. (E) mRNA analysis of siRNA silencing efficiency in organoid*^Mdr2KO liver^*. (F) Western blotting of siRNA silencing efficiency in organoid*^Mdr2KO liver^*. (G) Knockdown of TGR5 gene expression in organoid*^Mdr2KO liver^* significantly reduced the expression of Ki-67, TNF-α, and IL-6 (*n* = 4 for each group). (H) Western blotting of TGR5 in organoid*^Mdr2KO liver^*. Organoid*^Mdr2KO liver^* knocked down by SiTGR5_2 was cultured for 48 h, co-cultured with hP-MSCs for 24 h, and then stimulated with CXCL1. *P < 0.05, **P < 0.01, not significant (ns) > 0.05.

Western blotting analysis was used to determine TGR5, phosphatidylinositol 3 kinase (PI3K), p-PI3K, extracellular signal-regulated protein kinase (ERK), p-ERK, and β-actin protein expression in organoid*^Mdr2KO liver^*. hP-MSC treatment increased the phosphorylation of PI3K and ERK proteins, indicating that hP-MSCs improved the proliferation of bile duct cells via the TGR5/PI3K/ERK pathway (Fig. 8A).

Western blotting was used to detect TGR5, Pellino3, p-p65, p65, and β-actin protein expression in organoid*^Mdr2KO liver^*, organoid*^Mdr2KO liver^* after CXCL1/2 stimulation, and organoid*^Mdr2KO liver^* co-cultured with hP-MSCs after CXCL1/2 stimulation. Levels of Pellino3 protein increased and p65 phosphorylation decreased after hP-MSC treatment, indicating that hP-MSCs improved the cholangitis phenotype via the Pellino3/nuclear factor κB (NF-κB) pathway (Fig. 8B). To determine the interaction between tPellino3 and TGR5 in vivo, the liver tissues of Mdr2^+/+^, Mdr2^−/−^, and hP-MSC-treated Mdr2^−/−^ mice were subjected to co-immunoprecipitation (co-IP) analysis of endogenous TGR5 and Pellino3 proteins (Fig. 8C and D). We further speculated that hP-MSCs may increase the accumulation of Pellino3 protein by acting on TGR5 upstream of the Mdr2^−/−^ mice signaling axis, thereby affecting the transcription of NF-κB to alleviate liver inflammation.

Next, siRNA was used to knock down TGR5 expression in organoid*^Mdr2KO liver^*. Western blotting and qPCR analyses were performed to determine the knockdown efficiency of the 3 siRNA sequences used here, and the results showed that SiTGR5_1 and SiTGR5_2 significantly reduced TGR5 expression at the protein and mRNA levels (Fig. 8E and F). The effects on the downstream inflammatory and proliferative phenotype were examined, and the results showed that SiTGR5_2 was the most consistent with expectations (Fig. 8G). In addition, the organoid*^Mdr2KO liver^* with SiTGR5_2 knockdown was cultured for 48 h, co-cultured with hP-MSCs for 24 h, and then stimulated with CXCL1. Western blotting showed that the expression of TGR5 first decreased, then increased, and later decreased; the ERK pathway showed the same trend (Fig. 8H).

### hP-MSC treatment ameliorated the pathology of cholangiocytes in the human organoid model via TGR5/PI3K/ERK and TGR5/Pellino3/NF-κB signaling

**Fig. 9.**
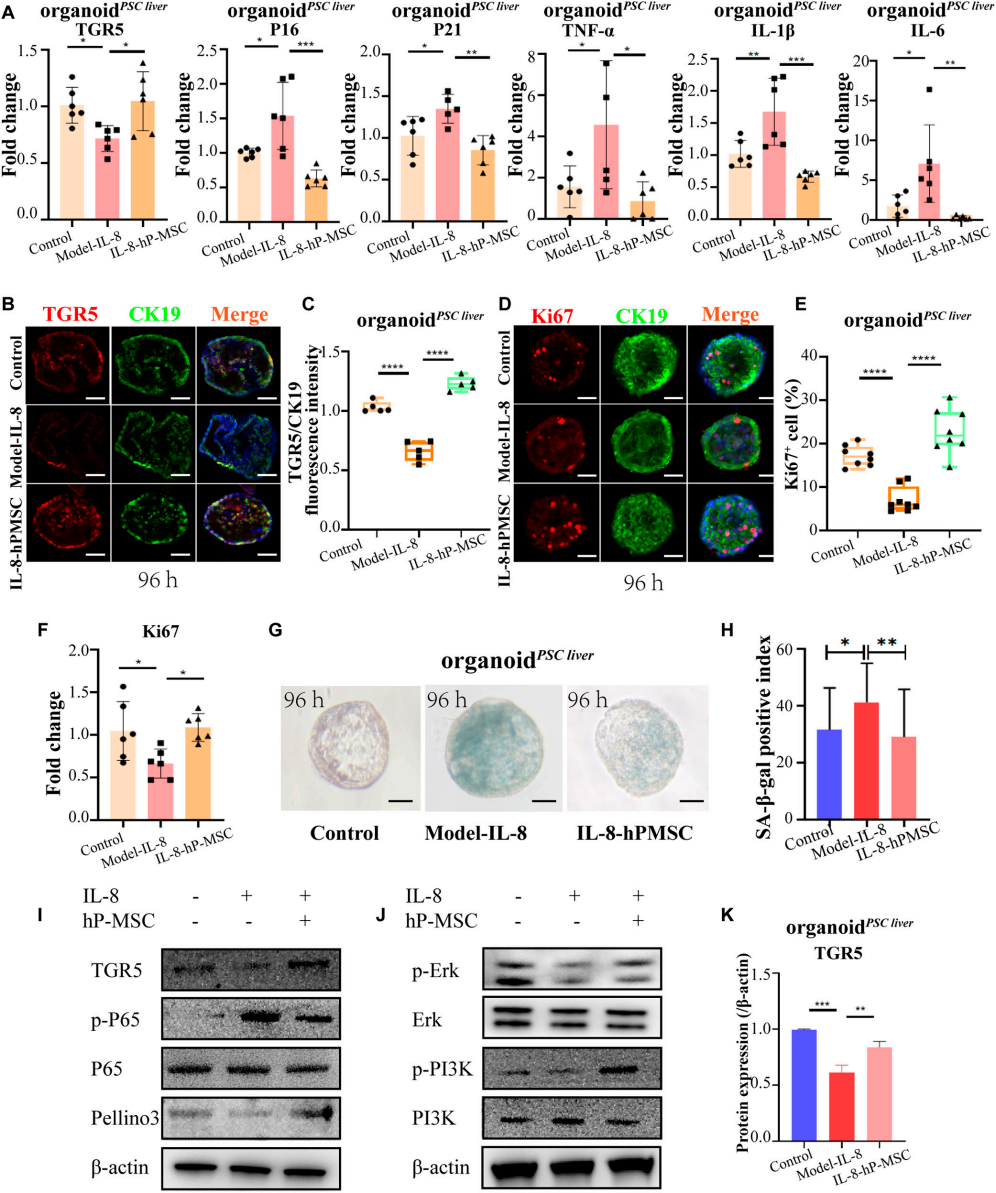
hP-MSC treatment ameliorated the pathological process of sclerosing cholangitis via the TGR5/PI3K/ERK and TGR5/Pellino3/NF-κB pathways in the human intrahepatic cholangiocyte organoid model. (A) TGR5, P16, P21, TNF-α, IL-1β, and IL-6 mRNA levels in organoid*^PSC liver^* were increased following stimulation with IL-8 (50 ng/ml) for 24 h, and the effects were ameliorated by co-culture with hP-MSCs for 24 h (*n* = 6 for each group). Organoid*^PSC liver^* was stimulated with IL-8, co-cultured with hP-MSCs, and double-labeled for (B) TGR5 and CK19 or (D) Ki-67 and CK19. Scale bar: 100 μm. (C) Fluorescence staining intensity of TGR5/CK19 in organoid*^PSC liver^* and corresponding quantification (*n* = 5 for each group). (E) Ki-67^+^ cell in organoid*^PSC liver^* and corresponding quantification (*n* = 8 for each group). (F) Ki-67 mRNA levels in organoid*^PSC liver^* were increased following stimulation with IL-8 (50 ng/ml) for 24 h, and the effects were ameliorated by co-culture with hP-MSCs for 24 h (*n* = 6 for each group). (G) SA-β-gal-positive organoids are shown. Organoid*^PSC liver^* was collected and analyzed at 96 h. Scale bar: 100 μm. (H) Percentages of SA-β-gal-positive organoids in 30 randomly selected areas. (I and J) Western blotting of TGR5, p-p65, p65, Pellino3, p-PI3K, PI3K, p-ERK, ERK, and β-actin protein levels in organoid*^PSC liver^*. (K) Quantificationof organoid*^PSC liver^* TGR5 protein normalized to β-actin by densitometry analysis. *P < 0.05, **P < 0.01, ***P < 0.001, ****P < 0.0001.

Organoid*^PSC liver^* was used to confirm whether IL-8 downregulated TGR5 and whether hP-MSCs had a rescue effect. Similar to co-culture analysis of organoid*^Mdr2KO liver^*, RT-qPCR was performed to determine mRNA levels in organoid*^PSC liver^*. Expression levels of the inflammatory marker TNF-α, IL-1β, and IL-6 and the senescence-associated markers p16^INK4a^ and p21^WAF1/Cip1^ were significantly reduced, and those of the proliferation markers Ki-67 were significantly increased in the hP-MSC-organoid*^PSC liver^*-IL-8 group compared with the organoid*^PSC liver^*-IL-8 group (Fig. 9A). Immunofluorescence analyses showed that TGR5 and Ki-67 expression were significantly decreased after IL-8 stimulation, while they were upregulated by hP-MSC treatment (Fig. 9B to E). Those of the proliferation markers Ki-67 were significantly increased in the hP-MSC-organoid*^PSC liver^*-IL-8 group compared with the organoid*^PSC liver^*-IL-8 group (Fig. 9F). Inhibition of senescence was demonstrated by senescence-associated (SA)-β-gal staining (Fig. 9G). The percentage of SA-β-gal-positive organoid*^PSC liver^* was also significantly decreased after co-culture with hP-MSCs (Fig. 9H). Western blotting analysis confirmed that hP-MSC treatment also ameliorated the inflammatory phenotype and inhibited the proliferation of organoid*^PSC liver^* through TGR5/PI3K/ERK and TGR5/Pellino3/NF-κB pathways (Fig. 9I to K).

## Discussion

The results of this study showed that TGR5 was differentially expressed in PSC and normal human cholangiocytes, and that IL-8 reduced the level of TGR5. These differences were preserved in organoid*^PSC liver^* and organoid*^Mdr2KO liver^*. hP-MSC treatment alleviated sclerosing cholangitis in BDL, DDC, and Mdr2^−/−^ mouse models. The effects of hP-MSC treatment in Mdr2^−/−^ mice were mainly mediated by secretion of IGF-1 and downregulation of CXCL1/2 in vivo, targeting TGR5 in cholangiocytes. The results in organoids showed that co-culture with hP-MSCs improved the proliferation potential of cholangiocytes via the TGR5/PI3K/ERK pathway, inhibited inflammation by binding of TGR5 and Pellino3, activated NF-κB expression, and decreased the levels of the inflammatory factors TNF-α, IL-1β, and IL-6. This research strategy will contribute to exploration of the pathological mechanism of PSC and the clinical application of MSCs in PSC patients.

During disease progression, PSC shows abnormal bile secretion, chronic inflammation, liver fibrosis, and other manifestations [16]. The bile acid regulatory receptors TGR5 and FXR have attracted a great deal of attention in research on cholestasis [17]. TGR5 is mainly localized in the primary cilia, apical plasma membrane, and nuclear membrane of bile duct cells [18]. Bile acid-induced activation of TGR5 regulates cell proliferation, triggers cytoprotective mechanisms, and promotes chloride secretion [19]. In the liver, TGR5 is expressed in nonparenchymal cells, including bile duct cells, sinusoidal endothelial cells, natural killer (NK) cells, and Kupffer cells [20–22]. Many studies have shown that the decline of TGR5 in PSC is specific, and is not seen in primary biliary cirrhosis and nonalcoholic fatty liver [23–25]. RNA-Seq analysis of liver tissue from PSC patients showed that TGR5 was not significantly increased, but was decreased apparently in cholangiocytes and increased apparently in Kupffer cells as determined by tyramide signal amplification (TSA) multiple immunofluorescence staining. RNA-Seq analysis also showed that TGR5 was apparently decreased in intrahepatic cholangiocyte organoid*^PSC liver^*. These results showed that TGR5 expression is specifically reduced in cholangiocytes of PSC patients.

As a 3-dimensional (3D) cell culture system, organoids can be used to study human development and disease progression [26]. Biliary immunity response and cholangiocyte senescence contribute to the pathogenesis of cholangitis, which are the most distinguishing features of PSC. Compared with liver organoids, the characteristics of intrahepatic cholangiocyte organoids can better reflect the pathological changes of PSC. Soroka et al. [8] reported that cholangiocyte organoids derived from PSC patients showed marked biliary senescence with upregulation of the senescence-associated secretory phenotype (SASP) markers p21 and SERPINE2 (but not other SASP-associated molecules, including IL-6 and IL-8), and proinflammatory mediators, including CCL20, HLA-DMA, and CD74. We constructed intrahepatic cholangiocyte organoids from human and mouse liver tissues. qPCR and RNA-Seq analyses showed that organoid*^PSC liver^* reflected the senescence and inflammatory phenotype of cholangiocytes, the 2 most important characteristics of PSC. Moreover, the differences between organoid*^healthy liver^* and organoid*^PSC liver^* mimicked those of the original liver tissues, indicating that they are good in vitro models.

There is still a lack of effective treatment for PSC. MSCs have entered phase II or III clinical trials for use in a number of conditions, including graft versus host disease [27], Crohn’s disease [28], and systemic lupus erythematosus [29]. Therapeutic strategies using MSCs may be applicable in patients with liver diseases. The therapeutic effects of MSCs are generally thought to be mainly due to their immunosuppressive effects and/or the release of nutritional factors, which are involved in cell regeneration and immune homeostasis. In the present study, based on evaluation of pathological sections, liver enzymes, total bile acids, and fibrosis, hP-MSC treatment delayed the progression of liver fibrosis and decreased inflammation in 3 mouse models of PSC. In addition, increased fluorescence was detected in the damaged liver in this study, indicating inflammatory chemotaxis of hP-MSCs. Surprisingly, hP-MSCs could differentiate into human hepatocyte-like cells in mice, although the number of these cells was very small.

PSC patients have disturbances in bile acid metabolism and inflammation, along with specifically reduced TGR5 expression in cholangiocytes. At present, little is known about the specific mechanisms underlying the effects of hP-MSC treatment in PSC. Therefore, we focused on the role of hP-MSCs in regulating bile acid metabolism and immune regulation. PSC patients have high serum IL-8 levels, which is a possible prognostic factor [30,31]. CXCL1/2, the mouse homologues of IL-8, were also increased apparently in the serum of Mdr2^−/−^ mice. Stimulation of organoid*^Mdr2KO liver^* with CXCL1/2 resulted in decreases in TGR5 mRNA and protein levels, respectively. Therefore, CXCL1/2 were considered to be TGR5 inhibitors. The decrease of TGR5 in organoid*^Mdr2KO liver^* by CXCL1/2 and the subsequent bile duct inflammatory response and weakening of proliferation capacity were ameliorated by co-culture with hP-MSCs. Next, we explored the specific signaling pathways downstream of TGR5 that may regulate the proliferation and inflammatory phenotype of cholangiocytes. Increased TGR5 expression activates the phosphorylation of PI3K, resulting in Ras activation. Activated Ras then activates Raf by binding its N-terminal domain. Raf, mitogen-activated protein kinase (MEK), and ERK are activated successively, and finally activated ERK enters the nucleus to increase cell proliferation. Liang et al. [32] reported that treatment with the TGR5 activator INT777 improved the neurological function of middle cerebral artery occlusion (MCAO) model mice. Colocalization of TGR5 and Pellino3, which can bind directly, increased after MCAO, apparently reducing Caspase-8 and NLRP3 levels. Pellino3 is a member of the mammalian Pellino family of E3 ubiquitin ligases [33]. To our knowledge, there have been no previous studies of Pellino3 in the liver. Our observations confirmed that TGR5 and Pellino3 bound directly to each other in the liver, and TGR5 activated Pellino3, which inhibited activation of NF-κB and decreased the levels of TNF-α, IL-1 β, IL-6, and other inflammatory factors. We also used IL-8 to damage organoid*^PSC liver^*, which was reversed by co-culture with hP-MSCs. IL-8 also reduced the proliferation and inflammatory phenotype of cholangiocytes in organoids. The effects of hP-MSCs were also mediated by the TGR5/PI3K/ERK and TGR5/Pellino3/NF-κB pathways.

MSC-derived IGF-1 has been shown to improve cell regeneration in a variety of disease models [34,35]. We confirmed that hP-MSCs secreted IGF-1 to stimulate the expression of TGR5 in organoid*^Mdr2KO liver^*, and could reverse the inflammatory and proliferative phenotypes.

Some deficiencies cannot be ignored for this study. (a) Some findings should be regarded as tentative and more studies with larger PSC liver samples will be required to confirm the results. For example, there may be marked differences in FXR between PSC and healthy control, as the sample size increases. (b) Despite all this, although an organoid system has various advantages, organoids still have some critical limitation for functional studies including the Matrigel-based system and single-cell-based organoids. A multi-organoid system platform for automated and continual in situ monitoring of organoid behaviors may be a feasible option.

## Materials and Methods

## Blood and liver sample collection from PSC patients and healthy controls

The study was performed in accordance with the Declaration of Helsinki. Four liver samples from 4 PSC patients who had undergone liver transplantation and 4 discarded tissues during donor liver repair as healthy controls were collected from Shulan (Hangzhou) Hospital. The sampling site was 5 cm away from the liver hilum and rich in intrahepatic cholangiocytes within 2 h after liver removal. Blood samples from 8 PSC patients and 35 healthy controls were obtained through The First Affiliated Hospital, Zhejiang University School of Medicine. The protocols using human tissues and blood samples were approved by the Research Ethics Committee of The First Affiliated Hospital, Zhejiang University School of Medicine (Approval No. 2021-158).

## hP-MSCs and biliary cholangitis mouse models

hP-MSCs were obtained from the Cell Bank of State Key Laboratory for the Diagnosis and Treatment of Infectious Diseases, Zhejiang University. Culture, phenotypic identification, and multilineage differentiation were performed as described previously [36]. All protocols using human tissues were approved by the Research Ethics Committee of The First Affiliated Hospital, Zhejiang University School of Medicine (Approval No. 2013-272). Cells at passages 3 to 5 were used for experiments.

Male (8 weeks old) homozygous Mdr2 gene knockout (Mdr2^−/−^) mice, 0.1% DDC diet-induced C57BL/6 mice, and BDL C57BL/6 mice were used as models of biliary cholangitis. Mdr2^−/−^ and Mdr2^+/+^ (FVB.129P2-Abcb4tm1Bor/J) mice were originally purchased from the Jackson Laboratory (Bar Harbor, ME, USA). C57BL/6 mice were purchased from Ziyuan Laboratory Animal Science and Technology Co. Ltd. (Hangzhou, China). All mice were maintained in ventilated cages under a 12-h light/dark cycle and fed with standard mouse chow and water. The animal study protocols were approved by the Animal Care Ethics Committee of The First Affiliated Hospital, Zhejiang University School of Medicine (Approval No. 2020-1088).

## Intrahepatic cholangiocyte organoids derived from human and mouse liver

The media used for organoid culture are listed in Table S2, including wash medium (WM), basal medium (BM), human liver expansion medium (h-EM), human liver isolation medium (h-IM), human liver digestion solution medium (h-DM), mouse liver expansion medium (EM), mouse liver isolation medium (IM), and mouse liver digestion solution medium (DM). The isolation and culture of intrahepatic cholangiocyte organoids from livers of adult Mdr2^−/−^ and Mdr2^+/+^ mice were performed as described previously [37,38]. Human liver tissues were washed twice with precooled WM and cut into pieces. The fragments were added to h-DM and digested at 37 °C in a shaking incubator for 1 to 1.5 h. After digestion, the supernatant was collected, filtered through a 70-μm filter (Falcon Plastics, Oxnard, CA, USA), and the filtrate was neutralized with WM. After centrifugation at 400 × *g* for 4 min, the particles were washed twice in BM. The final particles were resuspended in an appropriate amount of Matrigel (Corning, Bedford, MA, USA). Aliquots of 30 to 50 μl of the mixtures were plated into each well of 24-well cell culture plates (Corning) and incubated at 37 °C for 30 min to allow the Matrigel to polymerize. After polymerization, 500 μl of h-IM medium was added. Passage and medium exchange were performed as described for mouse organoids.

## RNA-Seq analysis

Total RNA of human liver tissues and human liver tissue-derived organoids were extracted using an RNAmini kit (Qiagen, Hilden, Germany). Organoid (passages 3 and 7) were cultured for 96 h and then were used to harvest RNA for RNA-sequencing (RNA-seq). Enrichment of mRNA, fragmentation, reverse transcription, library construction, sequencing using the Illumina NovaSeq 6000 platform (Illumina, San Diego, CA, USA), and data analysis were performed by Genergy Biotechnology Co. Ltd. (Shanghai, China). The raw data were processed with Skewer and data quality was checked with FastQC v0.11.2 (http://www.bioinformatics.babraham.ac.uk/projects/fastqc). The read length was 2 × 150 bp. Clean reads were aligned to the human genome hg38 assembly using STAR and StringTie. Transcript expression was calculated as FPKM (fragments per kilobase of exon model per million mapped reads) using Perl. Differentially expressed transcripts (DETs) between different time points (12 h vs. 0 h, 36 h vs. 0 h, 72 h vs. 0 h) were determined using the MA-plot-based method with the Random Sampling (MARS) model in the DEGseq package. Generally, in the MARS model, M = log2C1 − log2C2, and A = (log2C1 + log2C2)/2 (where C1 and C2 denote the counts of reads mapped to a specific gene obtained from 2 samples). The thresholds for determining DETs were *P* < 0.05 and absolute fold change ≥ 1. Then, DETs were chosen for function and signaling pathway enrichment analysis using the KEGG and GO databases. The significantly enriched pathways were determined when *P* < 0.05 and at least 2 affiliated genes were included.

## Bile acid detection by liquid chromatography/mass spectrometry

Liver tissues from 12-week-old Mdr2^+/+^, Mdr2^−/−^, and hP-MSC-treated Mdr2^−/−^ mice were used to detect bile acids. Bile acid contents were determined quantitatively by MetWare (http://www.metware.cn) using the AB Sciex QTRAP 6500 LC-MS/MS platform (Applied Biosystems, Foster City, CA, USA). Unsupervised PCA was performed using the prcomp function in R (www.r-project.org). The data were unit variance scaled before unsupervised PCA. Metabolites showing significant regulation among the 3 groups were determined by Variable important in projection (VIP) and absolute log2FC (fold change). VIP values were extracted from the results of orthogonal partial least squares discriminant analysis (OPLS-DA), which also included score plots and permutation plots generated using the MetaboAnalystR R package. The data were log transformed (log2) and mean centered before OPLS-DA. To avoid overfitting, a permutation test (200 permutations) was performed.

## Transfection of organoids with small interfering RNA

Organoids were transfected with 3 short siRNAs (50 nM) targeting TGR5. The organoids were cultured as described previously [39]. At 80% to 90% confluence, they were digested and collected, and single cells were resuspended in 450 μl of IM. The mixture was kept on ice until transfection. Transfection was performed by adding 50 μl of DMEM to each of 2 1.5-ml microcentrifuge tubes for each condition. Then, 1 μl of small interfering RNA (siRNA) targeting TGR5 (Gene Pharma, Shanghai, China) was added to one of the tubes and 4 μl of siRNA-Mate mixture was added to the other tube. Both tubes were incubated at room temperature for 5 min. The contents of the 2 tubes were mixed together and incubated at room temperature for a further 15 min. Then, 50 μl of DNA-siRNA-Mate mixture was added to 450 μl of single-cell suspension and this new mixture was transferred to one well of a 24-well plate, centrifuged in a prewarmed centrifuge at 37 °C at 600 × *g* for 1 h, and incubated at 37 °C for 2 to 4 h. After centrifugation at 400 × *g* for 4 min, the particles were washed twice in BM. The final particles were resuspended in an appropriate amount of Matrigel (Corning, Bedford, MA, USA). The sequences of 3 short siRNAs (50 nM) targeting TGR5 are shown in Table S3. siRNAs that showed significant knockdown effects were used in subsequent analyses.

## Statistical analysis

The data of all experiments are presented as the mean ± standard error of mean. The log-rank test was applied for survival analysis. Significance was determined by one-way analysis of variance (ANOVA) or 2-tailed Student’s *t* test. All statistical analyses were performed using GraphPad Prism 8 software (GraphPad Software, San Diego, CA). In all analyses, *P* < 0.05 was taken to indicate statistical significance.

Details of biliary cholangitis models of mice established by DDC and BDL, hP-MSC administration route and dosage, measurement of IL-8 and CXCL1/2, serum biochemical indexes, inflammatory cytokines and chemokines, Sirius red staining, Rhodamine 123 staining, co-culture of hP-MSCs and organoid, RNA extraction, real-time fluorescent qPCR analysis, immunofluorescence and immunohistochemical staining, TSA staining, flow cytometry, and Western blotting analysis were performed as described in the Supplementary Information. Stimulants used in the co-culture of hP-MSCs and organoids are listed in Table S4. The primer sequences used for tissue and organoid detection of human and mouse are listed in Table S5. The antibodies used in this experiment are all listed in Table S6.

## Data Availability

The data that support the findings of this study are available from the corresponding author upon reasonable request.

## Supplementary Materials

Supplementary 1 Table S1. Patient characteristics. Table S2. Human and mouse organoid medium formulations. Table S3. SiRNA sequences. Table S4. Stimulants used in the co-culture of hP-MSCs and organoids. Table S5. Primer sequences used for real-time PCR studies on tissues and organoids of human and mouse. Table S6. Antibody list. Fig. S1. Gene levels of liver tissues and organoids of human and mouse. Fig. S2. Construction of liver tissue-derived cholangiocyte organoid from Mdr2^−/−^ mice and Mdr2^+/+^ mice. Fig. S3. Hepatic fibrosis and pathological damage are the pathological consequence of PSC liver injury. Fig. S4. RNA-Seq analysis of the livers and organoids from PSC patients and healthy donors. Fig. S5. RNA-Seq analysis of liver tissue from PSC patients and healthy controls. Fig. S6. RNA-Seq analysis of organoids. Fig. S7. Transcriptome analysis revealed the cholangiocyte senescence-related, profibrotic cytokine-related, and angiogenesis in organoid*^PSC liver^*. Fig. S8. Surface markers of hP-MSCs determined by flow cytometry. Fig. S9. Detection of hP-MSC multi-differential potential in vitro. Fig. S10. hP-MSC treatment ameliorated the pathological process of sclerosing cholangitis BDL mouse models. Fig. S11. hP-MSC treatment by tail vein injection in Mdr2^−/−^ mice. Fig. S12. hP-MSC treatment by tail vein injection in Mdr2^−/−^ mice. Fig. S13. hP-MSC treatment ameliorated the pathological process of Mdr2^−/−^ mice. Fig. S14. Expression of TGR5 in liver tissues and organoids. Fig. S15. Enrichment of differential metabolites of bile acids. Fig. S16. hP-MSC treatment ameliorated bile acid metabolism in Mdr2^−/−^ mice. Fig. S17. Efficacy of hP-MSC treatment in the Mdr2^−/−^ mouse model of sclerosing cholangitis. Fig. S18. Expression of TGR5 in cholangiocytes in hP-MSC-treated DDC and BDL models. Fig. S19. Changes in mRNA expression in organoid^Mdr2KO liver^ co-cultured with hP-MSCs in vitro.
