# Supplementary material for: Human Placental Mesenchymal Stem Cells Relieve Primary Sclerosing Cholangitis via Upregulation of TGR5 in Mdr2−/− Mice and Human Intrahepatic Cholangiocyte Organoid Models
Source: Research (Wash D C). 2023 Aug 17;6:0207. doi: 10.34133/research.0207 (PMC10433880; doi:10.34133/research.0207)
Supplement: Supplementary 1 — Table S1. Patient characteristics. Table S2. Human and mouse organoid medium formulations. Table S3. SiRNA sequences. Table S4. Stimulants used in the co-culture of hP-MSCs and organoids. Table S5. Primer sequences used for real-time PCR studies on tissues and organoids of human and mouse. Table S6. Antibody list. Fig. S1. Gene levels of liver tissues and organoids of human and mouse. Fig. S2. Construction of liver tissue-derived cholangiocyte organoid from Mdr2−/− mice and Mdr2+/+ mice. Fig. S3. Hepatic fibrosis and pathological damage are the pathological consequence of PSC liver injury. Fig. S4. RNA-Seq analysis of the livers and organoids from PSC patients and healthy donors. Fig. S5. RNA-Seq analysis of liver tissue from PSC patients and healthy controls. Fig. S6. RNA-Seq analysis of organoids. Fig. S7. Transcriptome analysis revealed the cholangiocyte senescence-related, profibrotic cytokine-related, and angiogenesis in organoidPSC liver. Fig. S8. Surface markers of hP-MSCs determined by flow cytometry. Fig. S9. Detection of hP-MSC multi-differential potential in vitro. Fig. S10. hP-MSC treatment ameliorated the pathological process of sclerosing cholangitis BDL mouse models. Fig. S11. hP-MSC treatment by tail vein injection in Mdr2−/− mice. Fig. S12. hP-MSC treatment by tail vein injection in Mdr2−/− mice. Fig. S13. hP-MSC treatment ameliorated the pathological process of Mdr2−/− mice. Fig. S14. Expression of TGR5 in liver tissues and organoids. Fig. S15. Enrichment of differential metabolites of bile acids. Fig. S16. hP-MSC treatment ameliorated bile acid metabolism in Mdr2−/− mice. Fig. S17. Efficacy of hP-MSC treatment in the Mdr2−/− mouse model of sclerosing cholangitis. Fig. S18. Expression of TGR5 in cholangiocytes in hP-MSC-treated DDC and BDL models. Fig. S19. Changes in mRNA expression in organoidMdr2KO liver co-cultured with hP-MSCs in vitro. [file research.0207.f1.docx]

**Supplementary Information**

**Human Placental Mesenchymal Stem Cells Relieve Primary Sclerosing Cholangitis via Upregulation of TGR5 in Mdr2^−/−^ Mice and Human Intrahepatic Cholangiocyte Organoid Models**

Qigu Yao^1, 4^, Wenyi Chen^1, 4^, Yingduo Yu^1, 4^, Feiqiong Gao^1, 4^, Jiahang Zhou^1, 4^, Jian Wu^1, 4^, Qiaoling Pan^1, 4^, Jinfeng Yang^1, 4^, Lingling Zhou^1, 4^, Jiong Yu^1, 4^, Hongcui Cao*^1,3,4^, Lanjuan Li^1,2,4^

^1^State Key Laboratory for the Diagnosis and Treatment of Infectious Diseases, Collaborative Innovation Center for Diagnosis and Treatment of Infectious Diseases, The First Affiliated Hospital, Zhejiang University School of Medicine, 79 Qingchun Rd., Hangzhou City 310003, China

^2^ Jinan Microecological Biomedicine Shandong Laboratory, Jinan, Shandong 250117, China

^3^ Key Laboratory of Diagnosis and Treatment of Aging and Physic-chemical Injury Diseases of Zhejiang Province, 79 Qingchun Rd, Hangzhou City 310003, China

^4^ National Clinical Research Center for Infectious Diseases, Hangzhou, China

***Corresponding author:**

Hongcui Cao, M.D.

State Key Laboratory for the Diagnosis and Treatment of Infectious Diseases, The First Affiliated Hospital, Zhejiang University School of Medicine, 79 Qingchun Rd., Hangzhou City 310003, China. Tel: 86-571-87236451; Fax: 86-571-87236459

E-mail: [hccao@zju.edu.cn](mailto:hccao@zju.edu.cn)

**Sclerosing cholangitis mouse models**

**Mdr2 gene knock out (Mdr2^-/-^) mouse model**

Male Mdr2^-/-^ mice (8 weeks old) were randomly divided into two groups: 1) Mice injected with 100 µL phosphate-buffered saline (PBS) via the tail vein were regard as Model Group (n=30); 2) Mice injected with 5×10^5^ hP‐MSCs in 100 µL PBS were regard as Treatment Group (n=30); 3) Eight-week-old male Mdr2^+/+^ mice were as Control Group (n=30).

**BDL (Bile Duct Ligation) mouse model**

Wild type Male C57Bl/6 mice (8 weeks old) were used to perform the BDL (bile duct ligation) operation: after opening the peritoneal cavity of the mouse, the extrahepatic bile duct parallel to the portal vein was found and ligated with a surgical wire as described before(Gliedman et al., 1964). BDL mice injected with 5×10^5^ / 100 µL PBS containing hP‐MSCs as the Treatment group (Treatment); BDL mice received an equal volume of PBS were regard as Model group (Model), and the sham-operated group without surgical wire ligation were served as the control group (Control).

**DDC induced mouse model**

Wild type male C57Bl/6 mouse (6 weeks old) were feed with 0.1% 3,5-diethoxycarbonyl-1,4-dihydrocollidine (DDC) (Trophic Animal Feed High-tech Co. Ltd.) for 2 weeks to establish bile duct injury model. PBS injection via caudal vein were regard as Model group (Model); hP‐MSCs (5×10^5^ / 100 µL PBS) injection group were regard as Treatment Group (Treatment). Negative control mouse (Control) feed with normal chow diet.

After hP‐MSCs/PBS injection for 4 weeks, all mice (Control, Model, Treatment) were sacrificed. Serum were isolated and stored at −80°C. Partial liver sections were fixed in 4% paraformaldehyde and the remaining liver was frozen in liquid nitrogen for further experiments.

**In vivo tracking of hP-MSCs in mice**

hP-MSCs (5×10^5^ / 100 µL PBS) were labeled with 5 µM DiR as described in the instructions manual (D12731; ThermoFisher). Then, labeled hP-MSCs were injected via tail vein into Mdr2^-/-^ (8 weeks old). Mice injected with equal volume of PBS were regard as control. 3 hours later, all mice were sedated by isoflurane and imaged on an IVIS Spectrum (PerkinElmer), and living imaging were performed every 3 days then. 750 nm and 800 nm were selected as excitation and emission filters, respectively.

**Co-culture of hP-MSCs and organoids**

After organoid grew stably in the upper chamber for 2 days, Stimulants (200 ng/mL CXCL1, CXCL2) separately were added to the expansion medium (EM), and 1×10^5^ hP-MSCs were cultured in the lower chamber, respectively. After culturing for another 24h, removed the medium of hP-MSCs, co-culture the upper chamber with lower chamber and covered with 500 μL EM. Organoids in the control group grew only in EM, and medium in trans-well were replaced every 24 h. Stimulants are listed in the Table S1.

**The appearance time of primary organoids and the diameter of organoids**

The appearance time of primary intrahepatic cholangiocyte organoids derived from PSC liver (organoid*^PSC liver^*) and healthy donor liver (organoid*^healthy liver^*) was recorded by 40× objective and bright illumination. The diameter of primary organoids was detected and recorded by 40× objective and bright illumination of 30 randomly selected areas at day 8.

**ELISA assay for serum cytokines**

Collecting the whole blood of human, then centrifuged the samples at 20 ºC, 3500 rpm for 10 minutes to gained the serum. According to the manufacturer's agreement, human serum hIGF-1, hIL-8, and CXCL1/2 were measured by hIGF-1 set ELISA Kit (Beyotime biotech Co., Ltd., Shanghai, China), hIL-8 set ELISA Kit (Biolegend, San Diego, CA, USA), and CXCL1/2 set ELISA Kit (Cloud-Clone, Wuhan, China), separately.

**Immunofluorescence and immunohistochemical staining of human and murine liver derived organoids**

Immunofluorescence: human and mice liver derived organoids were collected and washed three times with pre-cold Basel Medium (BM) to remove Matrigel. After that, organoids were fixed with 4% (w/v) PFA for 1h, then permeabilized in 0.1% (v/v) Triton-X100 in PBS, and blocked for 1h in permeabilization buffer with 2% (v/v) fetal calf serum (FCS). Lastly, incubated the organoids overnight with primary antibodies in 24-well plate at 4 ℃ shaker. At the 2^nd^ day, the samples were washed three times (40 min/time）in PBS, then incubated with appropriate secondary antibodies for 2 hours. After incubation, organoids were washed again as previously mentioned. Mixed the organoids with PBS and counted the numbers. Then, organoids were observed and imaged via confocal laser scanning microscope (Zeiss LSM710; Carl Zeiss AG, Germany) on the same day using the appropriate laser intensity and exposure times to prevent thermally-induced sample movement.

The method of immunofluorescence quantitative analysis: Mean fluorescence intensity= IntDen / Area

Immunohistochemistry: organoids were washed with 1 × PBS and resuspended in 70% ethanol, then dehydrated and stained in 0.5% eosin ((w/v) 95% ethanol) for 30 minutes. After that, organoids were dehydrated in 100% ethanol and embedded in paraffin for further slides cutting. The following staining procedure were similar as the immunohistochemistry steps of paraffin slides. The first and second antibodies were listed as Table S2.

**Flow cytometry analysis of hp-MSCs**

Surface markers’ expression of hP‐MSCs was analyzed using flow cytometry. After washing by 0.5% bovine serum albumin (BSA) in PBS, hp-MSCs were incubated with allophycocyanin‐conjugated antibodies against human CD73, CD90, CD105, CD34, or CD45 (1:20; eBioscience Inc.,San Diego, CA) in darkness at room temperature for 30 min and then washed with PBS. Corresponding isotype antibodies (1:20; eBioscience Inc.) were used to eliminate nonspecific binding. The surface marker signals were detected by BD LSR II flow cytometer (Becton Dickinson Co., San Jose, CA).

**RNA isolation and qRT-PCR**

The Total RNA of mouse and human liver tissues and organoids were harvested using TRIzol reagent (Life Technologies Corporation, Carlsbad, CA), and complementary DNA (cDNA) was generated via HiScript III RT SuperMix for qPCR test (+gDNA wiper) (Vazyme, Nanjing, China). Then, qRT-PCR was performed with ChamQ Universal SYBR qPCR Master Mix (Vazyme) and analyzed by ABI 7500 Real-Time PCR System (Thermo Fisher Scientific, Waltham, MA, USA). The relative gene expression level was conducted with the 2–ΔΔCT method. Relevant primer sequences are listed in Table S4.

**Immunofluorescence (IF) and immunohistochemical (IHC) staining of liver tissues**

Frozen human and mouse liver sections (5µm) were used for immunofluorescence and immunohistochemical staining. Allow tissue to return to room temperature, then fixed with 4% (w/v) PFA for at least 1 day, embedded in paraffin and cut into slices (5µm). The following staining procedure was consistent with the routine IF/IHC staining procedure. The antibodies were referred to Table S5.

**Protein extraction and Western blot analysis**

The protein of liver tissue and organoids were extracted through RIPA lysis buffer (Beyotime Biotech Co., Ltd., Shanghai, China) (containing protease inhibitor cocktail and phosphatase inhibitor cocktail) (Sigma‐Aldrich) and treated by Ultrasonic Cell Disruptor (Bilang Co., Ltd., Shanghai, China). Western blotting was conducted as previously described(Feng et al., 2020), antibodies were listed in Table S5. Then, proteins were detected using PierceTM enhanced chemiluminescence (ECL) western blot analysis substrate (Thermo Fisher Scientific Inc.) and analyzed with ImageJ software (NIH, Bethesda, MD, United States).

**Tyramide Signal Amplification staining (TSA)**

The experimental procedure of multi-target immunofluorescence staining is similar to that of ordinary single label immunohistochemistry. The fluorescent dye in the TSA kit (AAT Bioquest, USA) can covalently bind the signal to the antigen under the action of HRP enzyme, and then the next round of single label staining can be connected until all the labels are completed, and then the DAPI can be stained again for observation. Experimental procedures were performed according to the instructions.

**Sirius Red staining**

Human and mouse liver samples were fixed in 4% paraformaldehyde, embedded in paraffin, and sectioned at 5 µm. After rehydration, the slides were stained with picrosirius red staining kit (Solarbio Inc., Beijing, China). The image was obtained with NanoZoomer 2.0-RS scanner (Hamamatsu Photonics, Hamamatsu, Japan). 2.9.

**Measurement of serum biochemical indexes and cytokines**

Serum aspartate aminotransferase (AST)，alanine aminotransferase (ALT), alkaline phosphatase (ALP) and total bile acid (TBIL) were detected by a dry chemistry analyzer (DRI-CHEM 4000ie; Fujifilm Corp., Tokyo, Japan).

According to the manufacturer’s instructions, serum inflammatory cytokines and chemokines were detected using LEGENDplexTM Multi-Analyte Flow Assay Kit (Biolegend, San Diego, CA, USA) and analyzed by CytoFLEX LX (Beckman Coulter, Brea, CA, USA).

**Rhodamine 123 staining**

To remove the Matrigel and gain organoids, culture medium was removed and covered with Cell Recovery Solution (Corning), then 24-well plate was placed on a 4℃ shaker and shake at 40-60 rpm/min vibrate horizontally for 0.5-1h. Then organoids were washed once with PBS. Diluting Rh123 mother liquor with expansion medium (EM) to prepare 20 µM Rh123 buffer. Then organoids pretreated with or without 10 μM verapamil (Catalog #V4629; Sigma-Aldrich) before added Rh123 buffer to the 24 well plate (500 µL/well) and incubated with organoids at 37 ℃ for 2 hours. After incubation, removed Rh123 buffer and washed the organoids with culture medium, then incubated with 4% (w/v) paraformaldehyde for 30 minutes. Lastly, washed organoids with PBS once and covered each well with 500 µL PBS. Organoids were observed and captured through fluorescence microscope with a fluorescein filter (ZOE ™ Fluorescence microscopic cell imager, Bio-Rad, USA).

**References：**

1. Feng, X., Liu, J., Xu, Y., Zhu, J., Chen, W., Feng, B., Pan, Q., Yu, J., Shi, X., Yang, J.*, et al.* (2020). Molecular mechanism underlying the difference in proliferation between placenta-derived and umbilical cord-derived mesenchymal stem cells. J Cell Physiol.
2. Gliedman, M.L., Girardet, R.E., Schwartz, A., Ryzoff, R., Lerner, B., and Karlson, K.E. (1964). HEPATIC VASCULAR ANATOMY AND MANOMETRY IN EXPERIMENTAL BILIARY OBSTRUCTION AND ASCITES. Surg Gynecol Obstet *119*, 749-757.

**Supplementary tables**

**Table S1 Patient Characteristics**

| **Patient** | **Diagnosis** | **Gender** | **Age**  **(year)** | **MELD** | **Spontaneous bacterial peritonitis** | **Cirrhosis** | **ALT**  **(U/L)** | **AST**  **(U/L)** | **TBil**  **(umol/L)** |
| --- | --- | --- | --- | --- | --- | --- | --- | --- | --- |
| P-01 | PSC | Female | 63 | 14 | NO | YES | 66 | 129 | 474 |
| P-02 | PSC | Male | 54 | 10 | NO | YES | 107 | 123 | 297 |
| P-03 | PSC | Female | 38 | 11 | NO | YES | 25 | 81 | 650 |
| P-04 | PSC | Male | 57 | 11 | NO | YES | 217 | 328 | 861 |

Abbreviations: MELD: model for end-stage liver disease; ALT: alanine aminotransferase, AST: aspartate aminotransferase; TBiL: total bilirubin; PSC: primary sclerosing cholangitis

**Table S2** **Human and mouse organoid medium formulations**

| **Wash medium (WM)** | | | |  |
| --- | --- | --- | --- | --- |
| **REAGENT or RESOURCE** | **DOSE** | **SOURCE** | **IDENTIFIER** |  |
| DMEM, high glucose | NA | Gibco | cat. no. 11995065 |  |
| FBS | 1% | Gibco | cat. no. 10099-141 |  |
| 1% penicillin/streptomycin | 1% | Gibco | cat. no. 2240826 |  |
| **Basal medium (BM)** | | | |  |
| **REAGENT or RESOURCE** | **DOSE** | **SOURCE** | **IDENTIFIER** | |
| Advanced DMEM/F-12 | NA | Gibco | cat. no. 12634010 | |
| Penicillin/streptomycin | 1% | Gibco | cat. no. 2240826 | |
| GlutaMAX | 1% | Gibco | cat. no. 35050-061 | |
| HEPES | 10 mM | Life Technologies | cat. no. 15630-05 | |
| **Human liver expansion medium (h-EM)** | | | | |
| **REAGENT or RESOURCE** | **DOSE** | **SOURCE** | **IDENTIFIER** | |
| Basal medium | NA | NA | NA | |
| B27 supplement (without vitamin A) | 1:50 | Life Technologies | cat. no. 12587-010 | |
| N2 supplement | 1:100 | Life Technologies | cat. no. 17502-048 | |
| *N*-acetylcysteine | 1 mM | Sigma-Aldrich | cat. no. A0737-5MG | |
| Rspo1-conditioned medium | 10% (vol/vol) | Home-made | NA | |
| nicotinamide | 10 mM | Sigma-Aldrich | cat. no. N0636 | |
| recombinant human [Leu15]-gastrin I 10 nM | 10 nM | Sigma-Aldrich | cat. no. G9145 | |
| recombinant human EGF | 50 ng/ml | Peprotech | cat. no. AF-100-15 | |
| recombinant human FGF10 | 100 ng/mL | Peprotech | cat. no. 100-26 | |
| recombinant human HGF | 25 ng/mL | Peprotech | cat. no. 100-39 | |
| Forskolin | 10 μM | Tocris Bioscience | cat. no. 1099 | |
| A83-01 | 5 μM | Tocris Bioscience | cat. no. 2939 | |
| **Human liver isolation medium (h-IM)** | | | | |
| **REAGENT or RESOURCE** | **DOSE** | **SOURCE** | **IDENTIFIER** | |
| Human expansion medium | NA | Home-made | NA | |
| recombinant human Noggin | 25 ng/mL | Peprotech | cat. no. 120-10C | |
| Wnt3a-conditioned medium | 30% (vol/vol) | Home-made | NA | |
| Rho kinase inhibitor (Y-27632) | 10 μM | Sigma-Aldrich | cat. no. Y0503 | |
| **Human liver digestion solution (h-DM)** | | | | |
| **REAGENT or RESOURCE** | **DOSE** | **SOURCE** | **IDENTIFIER** | |
| Earle’s Balanced Salt Solution | NA | Sigma-Aldrich | cat. no. H6648-500 | |
| DNaseI | 0.1 mg/mL | Sigma-Aldrich | cat. no. DN25 | |
| Collagenase D | 2.5 mg/mL | Roche | cat. no. 1108866001 | |
| Dispase II | 2.5 mg/mL | Life Technologies | cat. no. 17105-041 | |
| **Mouse liver expansion medium (EM)** | | | | |
| **REAGENT or RESOURCE** | **DOSE** | **SOURCE** | **IDENTIFIER** | |
| Basal medium | NA | NA | NA | |
| B27 supplement (without vitamin A) | 1:50 | Life Technologies | cat. no. 12587-010 | |
| *N*-acetylcysteine | 1 mM | Sigma-Aldrich | cat. no. A0737-5MG | |
| Rspo1-conditioned medium | 5% (vol/vol) | Home-made | NA | |
| nicotinamide | 10 mM | Sigma-Aldrich | cat. no. N0636 | |
| recombinant human [Leu15]-gastrin I 10 nM | 10 nM | Sigma-Aldrich | cat. no. G9145 | |
| recombinant mouse EGF | 50 ng/mL | Peprotech | cat. no. 315-09 | |
| recombinant human FGF10 | 100 ng/mL | Peprotech | cat. no. 100-26 | |
| recombinant human HGF | 50 ng/mL | Peprotech | cat. no. 100-39 | |
| **Mouse liver isolation medium (IM)** | | |  | |
| **REAGENT or RESOURCE** | **DOSE** | **SOURCE** | **IDENTIFIER** | |
| Mouse expansion medium | NA | Home-made | NA | |
| recombinant mouse Noggin | 25 ng/mL | Peprotech | cat. no. 250-38 | |
| Wnt3a-conditioned medium | 30% (vol/vol) | Home-made | NA | |
| Rho kinase inhibitor (Y-27632) | 10 μM | Sigma-Aldrich | cat. no. Y0503 | |
| **Mouse liver digestion solution (DM)** | | | | |
| **REAGENT or RESOURCE** | **DOSE** | **SOURCE** | **IDENTIFIER** | |
| Wash media | NA | Home-made | NA | |
| DNaseI | 0.1 mg/mL | Sigma-Aldrich | cat. no. DN25 | |
| Collagenase D | 0.125 mg/mL | Roche | cat. no. 1108866001 | |
| Dispase II | 0.125 mg/mL | Life Technologies | cat. no. 17105-041 | |

**Table S3 SiRNA sequences**

| **Name** | **Sequence（5'-3'）** |
| --- | --- |
| TGR5(m)-si-1 | CUUCCUAAGCCUACUACUA |
|  | UAGUAGUAGGCUUAGGAAG |
| TGR5 (m)-si-2 | GAACUCUGUUAUCGCUCAU |
|  | AUGAGCGAUAACAGAGUUC |
| TGR5 (m)-si-3 | GCAUUGACCUGGACUUGAA |
|  | UUCAAGUCCAGGUCAAUGC |

**Table S4** **Stimulants used in the co-culture of hP-MSCs and organoids**

| **REAGENT or RESOURCE** | **DOSE** | **SOURCE** | **IDENTIFIER** |
| --- | --- | --- | --- |
| TNF-α | 50ng/mL | Peprotech | cat. no. 315-01A |
| IFN-γ | 50ng/mL | Peprotech | cat. no. 315-05 |
| HGF | 50ng/mL | Peprotech | cat. no. 100-39 |
| IGF-1 | 50ng/mL | Peprotech | cat. no. AF-100-11 |
| PGE2 | 50ng/mL | Bio-Techne | cat. no. 2296 |
| IL-8 | 50ng/mL | Peprotech | cat. no. 200-08 |
| CXCL1 | 200ng/mL | Peprotech | cat. no. 250-11 |
| CXCL2 | 200ng/mL | Peprotech | cat. no. 250-15 |

**Table S5** **Primer sequences used for Real-time PCR studies on tissues and organoids of human and mouse**

| **Primer** | **Sequence** |
| --- | --- |
| Fxr (F) | TGTGAGGGCTGCAAAGGTTT |
| Fxr (R) | ACATCCCCATCTCTCTGCAC |
| Cyp7a1 (F) | TGGAATAAGGAGAAGGAAAGTA |
| Cyp7a1 (R) | TGTGTCCAAATGCCTTCGCAGA |
| Cyp27b1 (F) | TCCTGGCTGAACTCTTCTGC |
| Cyp27b1 (R) | CCAGACCATATTGGCCCGTA |
| MRP2 (F) | GCTTCCCATGGTGATCTCTT |
| MRP2 (R) | ATCATCGCTTCCCAGGTACT |
| TGF-β1 (F) | CTCCCGTGGCTTCTAGTGC |
| TGF-β1 (R) | GCCTTAGTTTGGACAGGATCTG |
| PCNA (F) | TTTGAGGCACGCCTGATCC |
| PCNA (R) | GGAGACGTGAGACGAGTCCAT |
| α-SMA (F) | TGCTGACAGAGGCACCACTGAA |
| α-SMA (R) | CAGTTGTACGTCCAGAGGCATAG |
| Ki67 (F) | GAGGAGAAACGCCAACCAAGAG |
| Ki67 (R) | TTTGTCCTCGGTGGCGTTATCC |
| TGR5 (F) | CACTGCTCTTCTTGCTGTGTTGG |
| TGR5 (R) | GAGCGATAACAGAGTTCCAGGC |
| Collagen I (F) | TCCTCCAGGGATCCAACGA |
| Collagen I (R) | GGCAGGCGGGAGGTCTT |
| Timp-1 (F) | GCATCTCTGGCATCTGGCATC |
| Timp-1 (R) | GGTATAAGGTGGTCTCGTTGA |
| LGR5 (F) | AGAGCCTGATACCATCTGCAAAC |
| LGR5 (R) | TGAAGGTCGTCCACACTGTTGC |
| CK19 (F) | AATGGCGAGCTGGAGGTGAAGA |
| CK19 (R) | CTTGGAGTTGTCAATGGTGGCAC |
| SOX9 (F) | CACACGTCAAGCGACCCATGAA |
| SOX9 (R) | TCTTCTCGCTCTCGTTCAGCAG |
| ALB (F) | CAGTGTTGTGCAGAGGCTGACA |
| ALB (R) | GGAGCACTTCATTCTCTGACGG |
| HNF4a (F) | TGCGAACTCCTTCTGGATGACC |
| HNF4a (R) | CAGCACGTCCTTAAACACCATGG |
| IL-6 (F) | TACCACTTCACAAGTCGGAGGC |
| IL-6 (R) | CTGCAAGTGCATCATCGTTGTTC |
| TNF-α (F) | GGTGCCTATGTCTCAGCCTCTT |
| TNF-α (R) | GCCATAGAACTGATGAGAGGGAG |
| IL-1β (F) | TGGACCTTCCAGGATGAGGACA |
| IL-1β (R) | GTTCATCTCGGAGCCTGTAGTG |
| P16 (F) | TGTTGAGGCTAGAGAGGATCTTG |
| P16 (R) | CGAATCTGCACCGTAGTTGAGC |
| P21 (F) | TCGCTGTCTTGCACTCTGGTGT |
| P21 (R) | CCAATCTGCGCTTGGAGTGATAG |
| h-LGR5 (F) | CCTGCTTGACTTTGAGGAAGACC |
| h-LGR5 (R) | CCAGCCATCAAGCAGGTGTTCA |
| h-CK19 (F) | AGCTAGAGGTGAAGATCCGCGA |
| h-CK19 (R) | GCAGGACAATCCTGGAGTTCTC |
| h-SOX9 (F) | AGGAAGCTCGCGGACCAGTAC |
| h-SOX9 (R) | GGTGGTCCTTCTTGTGCTGCAC |
| h-ALB (F) | GATGAGATGCCTGCTGACTTGC |
| h-ALB (R) | CACGACAGAGTAATCAGGATGCC |
| h-HNF4a (F) | GGTGTCCATACGCATCCTTGAC |
| h-HNF4a (R) | AGCCGCTTGATCTTCCCTGGAT |
| h-TGR5 (F) | GCTGCTTCTTCCTGAGCCTACT |
| h-TGR5 (R) | TTGGGAGCCAAGTAGACGAGGA |
| h-Ki67 (F) | GAAAGAGTGGCAACCTGCCTTC |
| h-Ki67 (R) | GCACCAAGTTTTACTACATCTGCC |
| h-PCNA (F) | CAAGTAATGTCGATAAAGAGGAGG |
| h-PCNA (R) | GTGTCACCGTTGAAGAGAGTGG |
| h-TGF-β1(F) | TACCTGAACCCGTGTTGCTCTC |
| h-TGF-β1 (R) | GTTGCTGAGGTATCGCCAGGAA |
| h-IL-6 (F) | AGACAGCCACTCACCTCTTCAG |
| h-IL-6 (R) | TTCTGCCAGTGCCTCTTTGCTG |
| h-TNF-α (F) | CTCTTCTGCCTGCTGCACTTTG |
| h-TNF-α (R) | ATGGGCTACAGGCTTGTCACTC |
| h-IL-1β (F) | CCACAGACCTTCCAGGAGAATG |
| h-IL-1β (R) | GTGCAGTTCAGTGATCGTACAGG |
| h-P16 (F) | CTCGTGCTGATGCTACTGAGGA |
| h-P16 (R) | GGTCGGCGCAGTTGGGCTCC |
| h-P21 (F) | AGGTGGACCTGGAGACTCTCAG |
| h-P21 (R) | TCCTCTTGGAGAAGATCAGCCG |
| h-GAPDH (F) | GTCTCCTCTGACTTCAACAGCG |
| h-GAPDH (R) | ACCACCCTGTTGCTGTAGCCAA |

**Table S6 Antibody list**

| **Primary antibody** | | | | | |
| --- | --- | --- | --- | --- | --- |
| **Antibody** | **Source** | **Product code** | **Species** | **Dilution** | |
| SOX9 | Abcam | ab185966 | Rabbit IgG | 100(IF) | |
| EPCAM | Proteintech | 66316-1-lg | Mouse IgG1 | 400(IF) | |
| CYP3A4 | Santa Cruz | sc-53850 | Mouse IgG1 | 100(IF) | |
| Cytokeratin 19 | Abcam | ab194399 | Mouse IgG1 | 100(IF) | |
| Cytokeratin 7 | Abcam | ab181598 | Rabbit IgG | 100(IF) | |
| Cytokeratin 19 | Abcam | ab52625 | Rabbit IgG | 400(IHC) | |
| Albumin | Abcam | ab2406 | Rabbit IgG | 500(IHC) | |
| PCNA | Abcam | ab92552 | Rabbit IgG | 100(IF) | |
| p16INK4a | Abcam | ab54210 | Mouse IgG2b | 100(IF); 200(IHC) | |
| p21 | Abcam | ab188224 | Rabbit IgG | 100(IF); 200(IHC) | |
| p21 | Abcam | ab109520 | Rabbit IgG | 100(IF); 200(IHC) | |
| Ki67 | Abcam | ab16667 | Rabbit IgG | 100(IF) | |
| α-SMA | servicebio | GB111364 | Rabbit IgG | 200(IHC) | |
| Beta actin | proteintech | 66009-1-Ig | Mouse IgG1 | 2000 (WB) | |
| PI3 Kinase p85 | Cell Signaling Technology | #4257 | Rabbit IgG | 1000 (WB) | |
| P-PI3 Kinase p85 | Affinit | #AF3241 | Rabbit IgG | 2000 (WB) | |
| NF-κB p65 | Cell Signaling Technology | #8242 | Rabbit IgG | 1000 (WB) | |
| P-NF-κB p65 | Cell Signaling Technology | #3033 | Rabbit IgG | 2000 (WB) | |
| p44/42 | Cell Signaling Technology | #4695 | Rabbit IgG | 2000 (WB) | |
| P-p44/42 | Cell Signaling Technology | #4370 | Rabbit IgG | 2000 (WB) | |
| TGR5 | Abcam | ab72608 | Rabbit IgG | 50(IF); 1000 (WB); 100(IHC) | |
| Pellino 3 | Santa Cruz | sc-376466 | Mouse IgG1 | 50(IP); 500(WB) | |
| **Conjugated antibody** | | | | | |
| **Antibody** | | **Company** | **Code number** | | **Ig species** |
| PE HLA-DR | | Biolegend | 307605 | | Mouse IgG1, κ |
| PE CD90 | | Biolegend | 328109 | | Mouse IgG1, κ |
| PE CD34 | | Biolegend | 343505 | | Mouse IgG1, κ |
| PE CD11b | | Biolegend | 301305 | | Mouse IgG1, κ |
| PE CD105 | | Biolegend | 323205 | | Mouse IgG1, κ |
| PE CD45 | | Biolegend | 304007 | | Mouse IgG1, κ |
| PE CD13 (WM-15) | | ebiocsience | 12-0138-42 | | Mouse IgG1, κ |
| PE CD19 | | Biolegend | 302207 | | Mouse IgG1, κ |
| PE CD29 (TS2/16) | | ebiocsience | 12-0299-42 | | Mouse IgG1, κ |
| PE CD73 (Ecto-5'-nucleotidase) | | Biolegend | 344003 | | Mouse IgG1, κ |
| PE Isotype Ctrl Antibody | | Biolegend | 400112 | | Mouse IgG1, κ |
| **Secondary antibody** | | | | | |
| **Secondary antibody** | | **Company** | **Code number** | | **Dilution** |
| Goat Anti-Rabbit IgG H&L (HRP) | | Abcam | ab6721 | | 400 |
| Goat anti-Mouse IgG (H+L) Cross-Adsorbed Secondary Antibody, Alexa Fluor 488 | | ThermoFisher | A-11001 | | 400 |
| Rabbit Anti-Mouse IgG H&L (HRP) | | Abcam | ab6728 | | 400 |
| Goat Anti-Rabbit IgG H&L (Alexa Fluor® 647） | | Abcam | ab150083 | | 400 |
| Goat Anti-Mouse IgG H&L (Alexa Fluor® 555) | | Abcam | ab150114 | | 400 |

**Supplementary figures and figure legends**


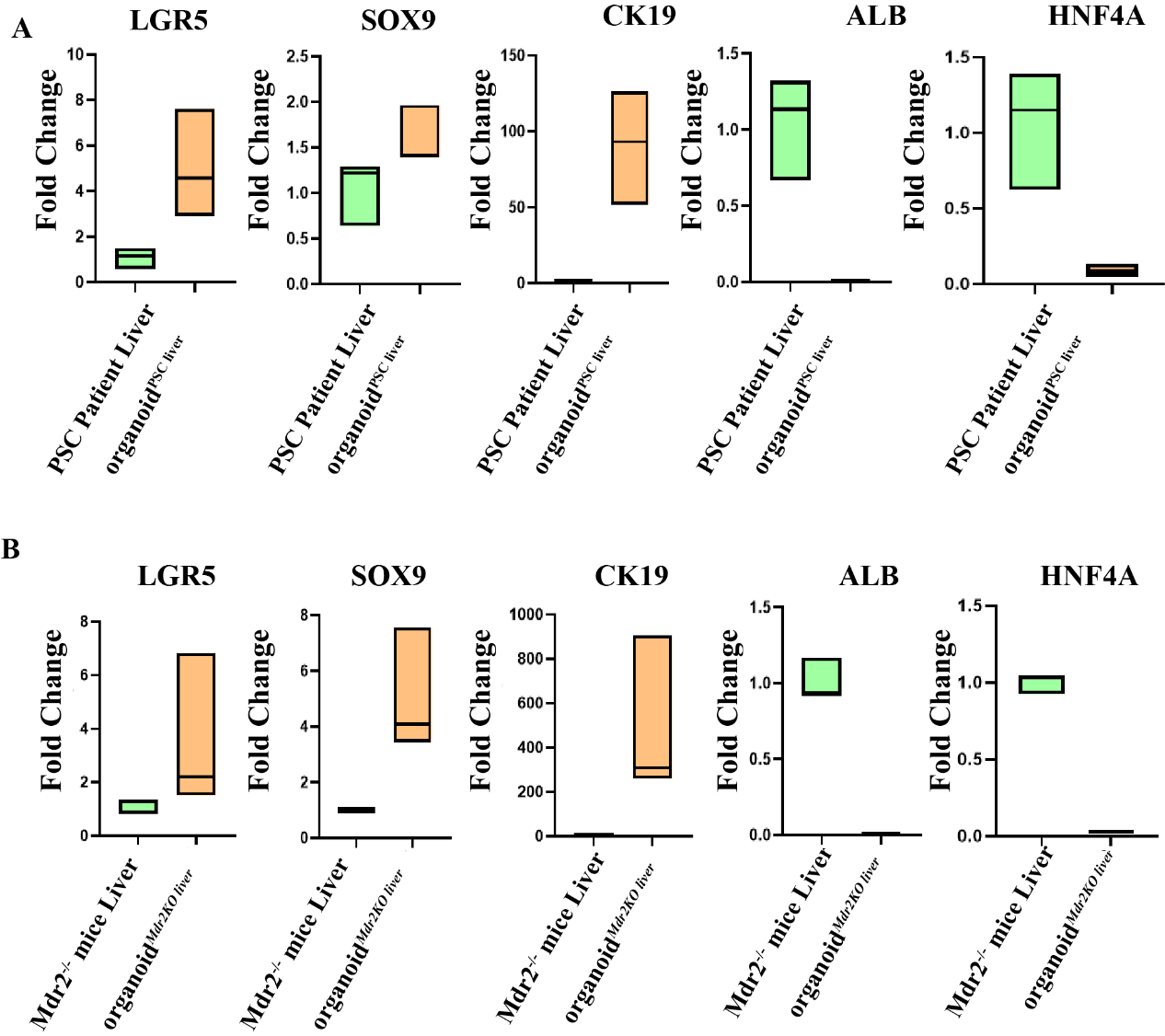


**Figure S1. Gene levels of liver tissues and organoids of human and mouse.**

**(A)** Comparison the gene levels of a bile duct cell marker (CK19), stemness markers (SOX9 and LGR5), and liver markers (ALB and HNF4A) in organoid*^PSC liver^* and liver tissue of PSC patients by real-time fluorescent quantitative PCR (qPCR). **(B)** Comparison the gene levels of a bile duct cell marker (CK19), stemness markers (SOX9 and LGR5), and liver markers (ALB and HNF4A) in organoid*^Mdr2KO liver^* and liver tissue of Mdr2^−/−^ mice by qPCR.


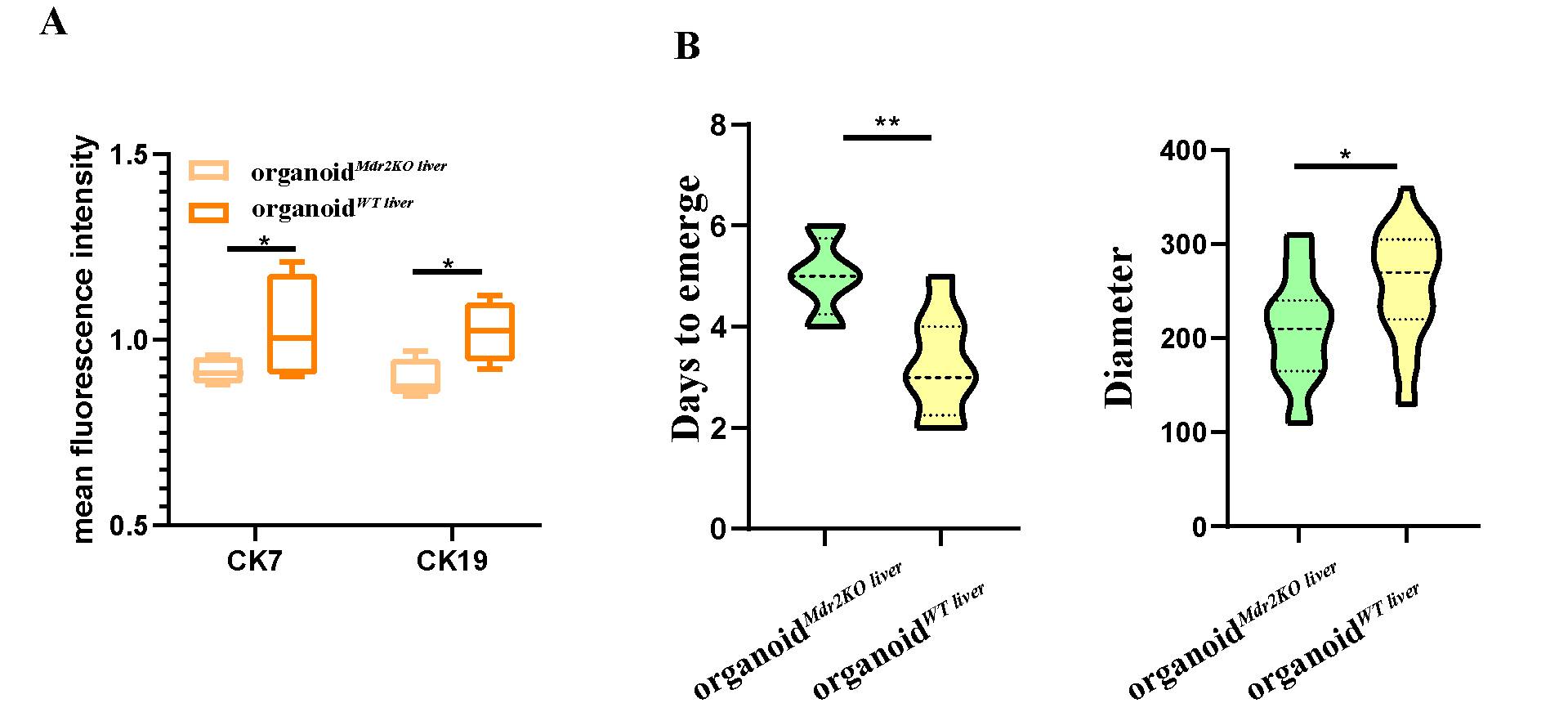


**Figure S2. Construction of liver tissue derived** **cholangiocyte organoid from Mdr2^−/−^ mice and Mdr2^+/+^ mice. (A)** Quantitative analysis of CK7, CK19 fluorescence intensity between organoid*^healthy liver^* and organoid*^PSC liver^*. **(B)** The appearance time of primary intrahepatic cholangiocyte organoids derived Mdr2^−/−^ mice (organoid^Mdr2KO liver^) and Mdr2^+/+^ mice liver (organoid*^WT liver^*), and the diameter of organoid on day 8. **P* < 0.05, ***P* < 0.01.


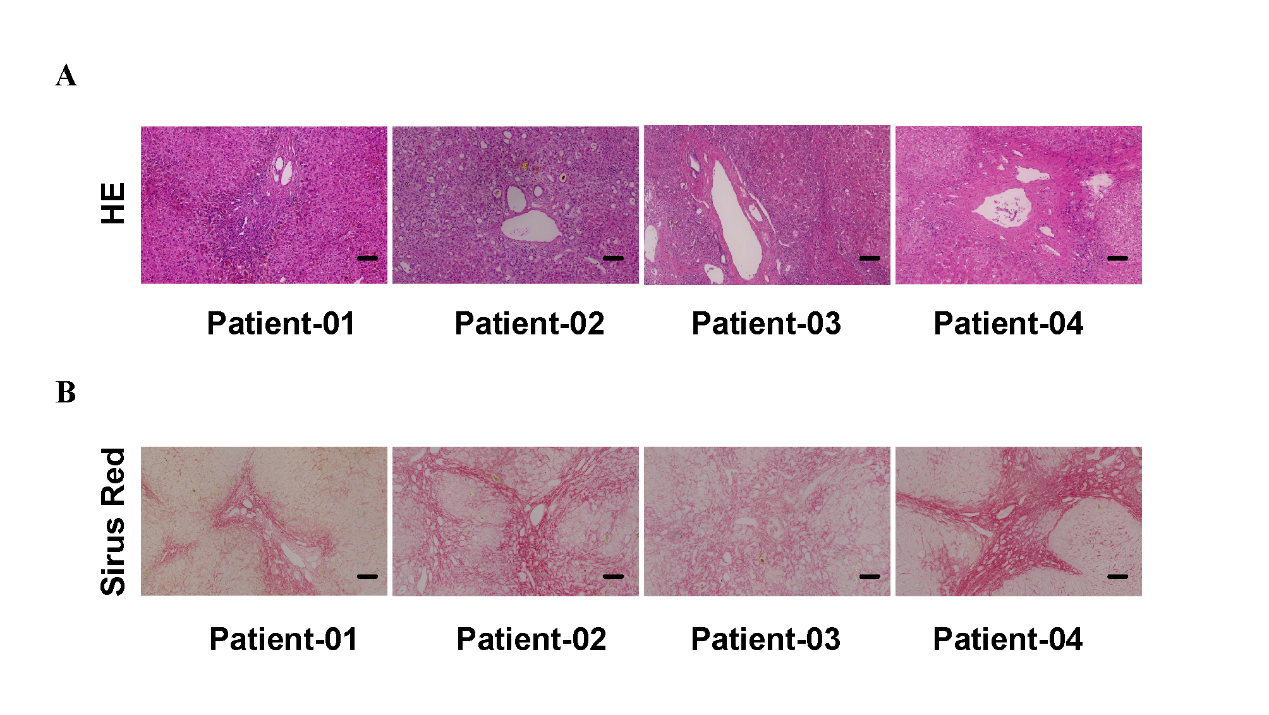


**Figure S3. Hepatic fibrosis and pathological damage are the pathological consequence of PSC liver injury.**

**(A)** HE staining revealed robust liver damage in 4 PSC patients. Scale bar: 100 μm. **(B)** Sirius Red stainin indicated collagenous matrix formation in 4 PSC patients. Scale bar: 100 μm.


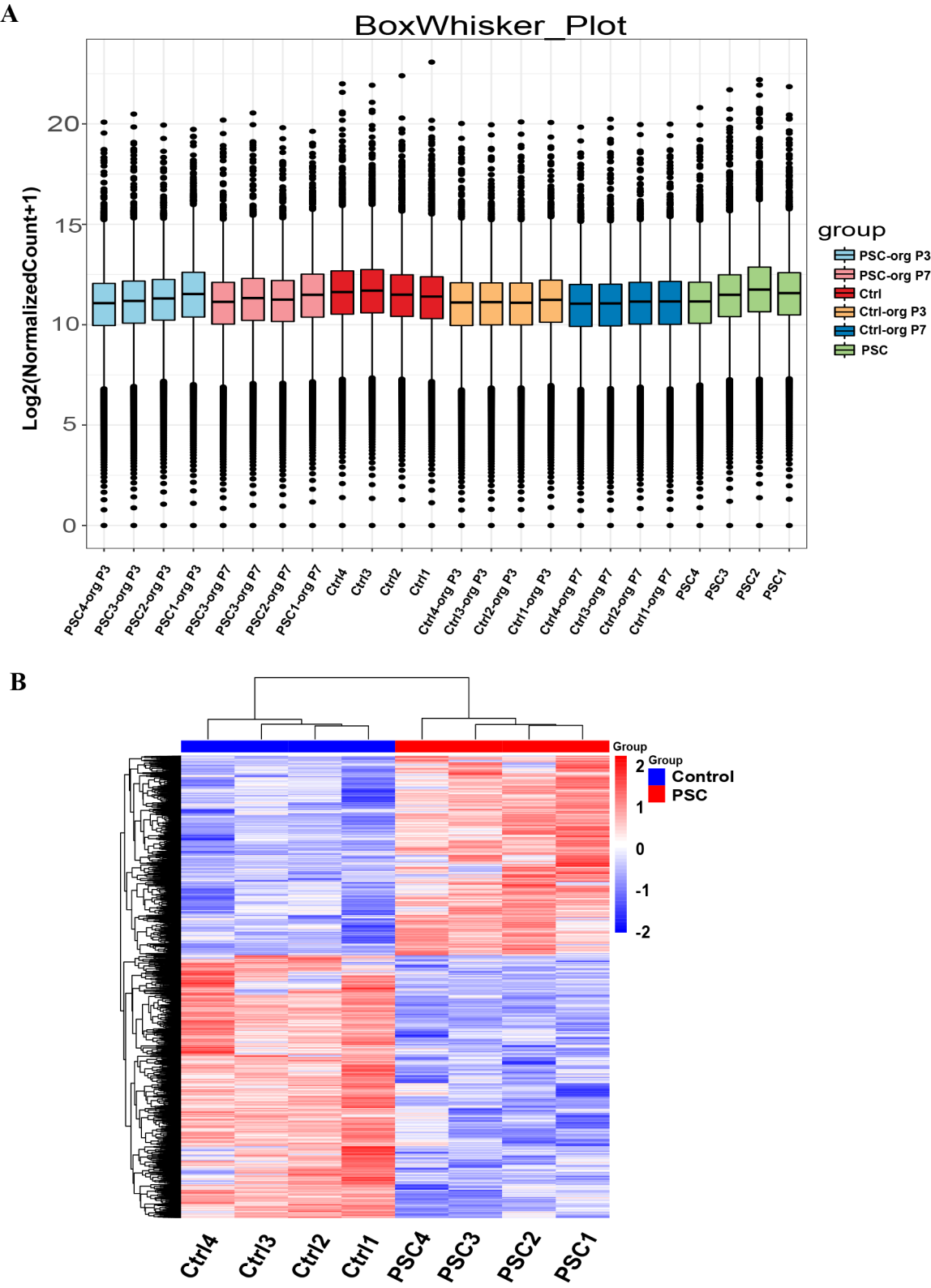


**Figure S4. RNA-Seq analysis of the livers and organoids from PSC patients and healthy donors.**

**(A)** Box whisker plot showing 24 cases (4 PSC liver tissue and 4 healthy control liver tissue and organoid from 8 PSC liver tissue in passage 3,7 and 8 healthy control liver tissue in passage 3,7) of normalized raw data. **(B)** Hierarchical clustering was performed to determine the overall differences between PSC and healthy control liver tissue.


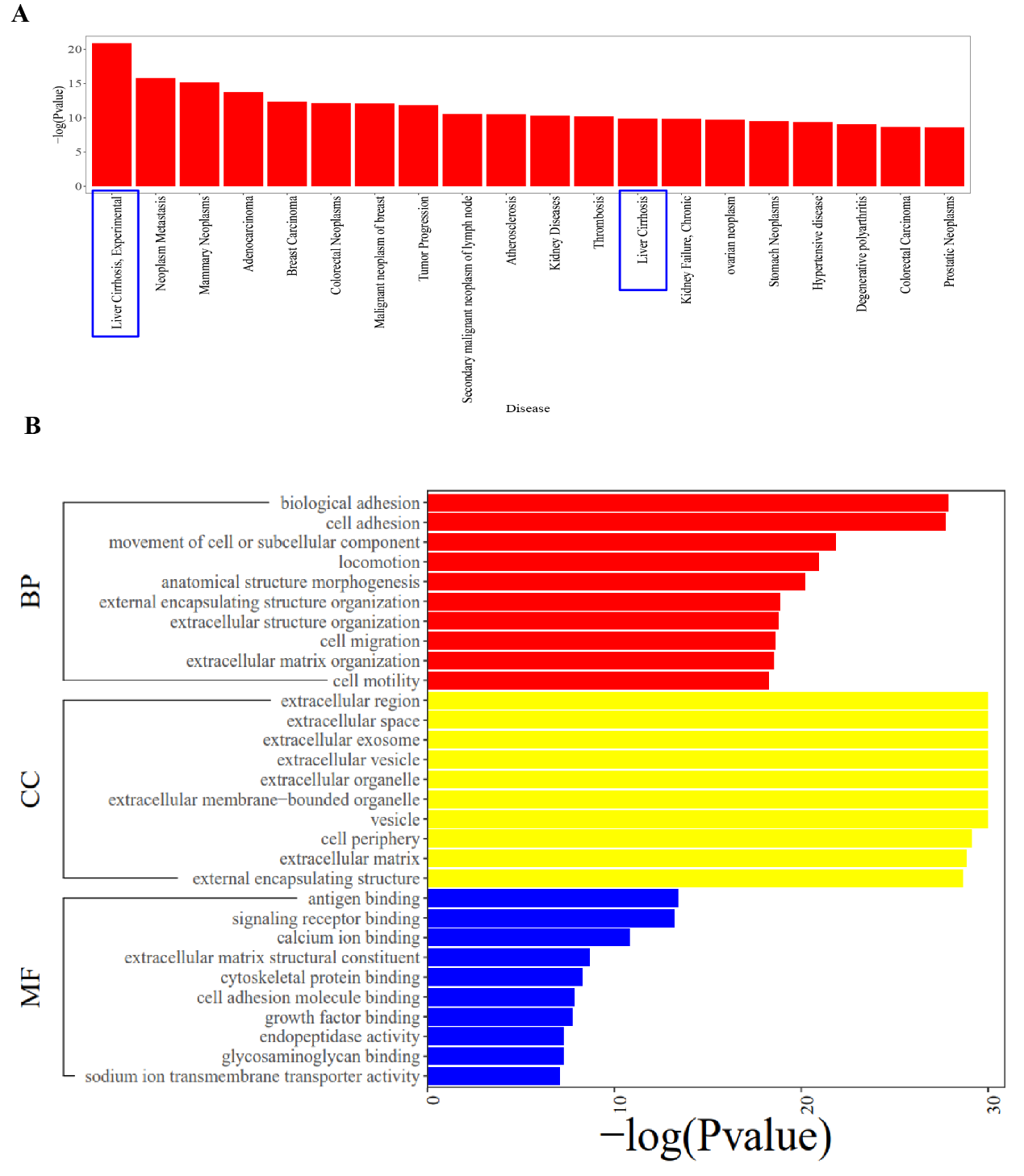


**Figure S5. RNA-Seq analysis of liver tissue from PSC patients and healthy controls.**

**(A)** Disease enrichment analysis of sets of differentially expressed genes (DEGs) from 4 PSC patients and 4 healthy controls. **(B)** Gene Ontology enrichment analysis of DEGs in biological process (BP), molecular function (MF), and cellular component (CC).


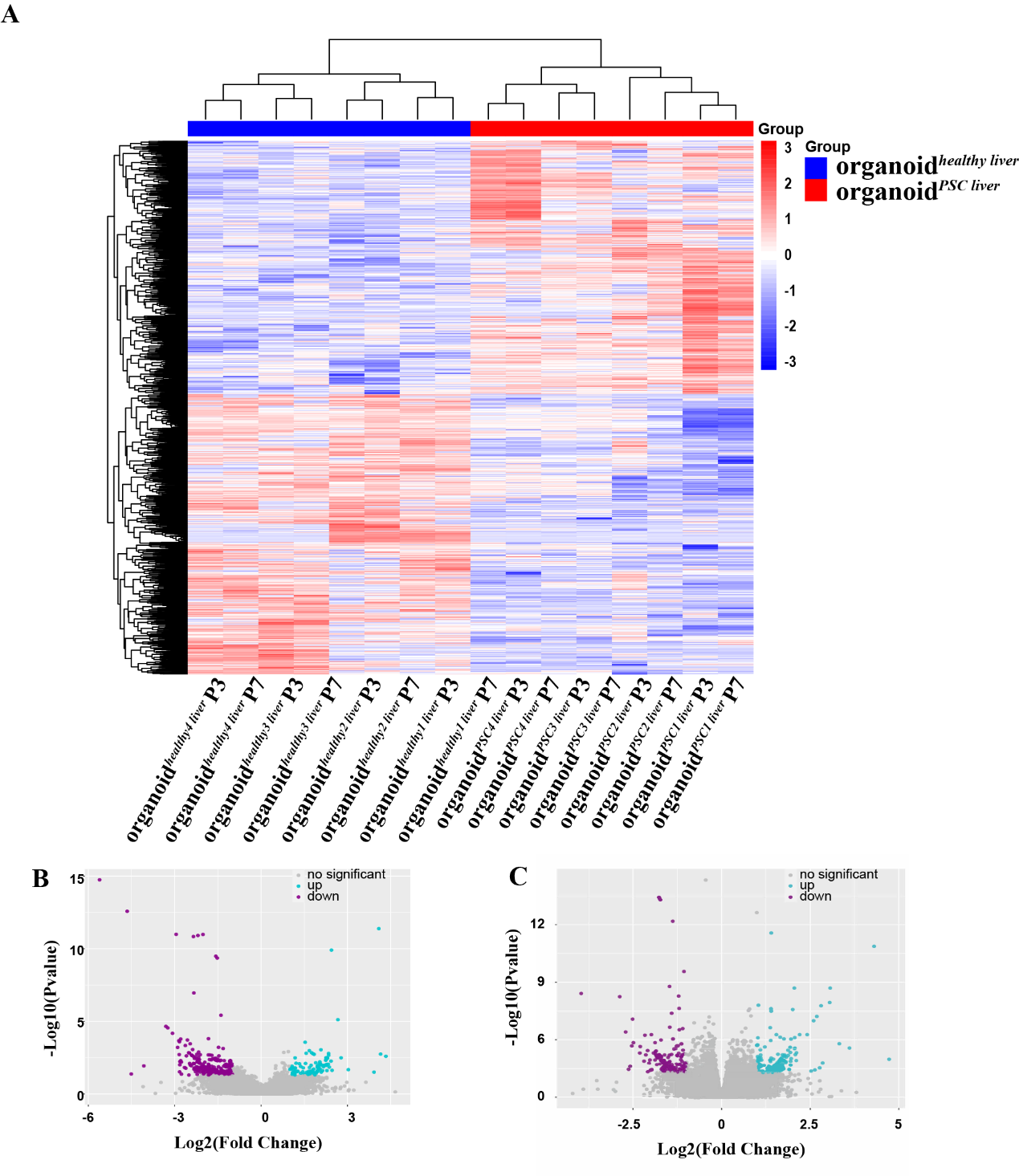


**Figure S6. RNA-Seq analysis of organoids.**

1. Hierarchical clustering was performed to determine the overall differences between organoid*^PSC liver^* and organoid*^healthy liver^* (passage 3 and passage 7, *n* = 16). **(B)** Volcano plot of organoid differentially expressed genes (DEGs). Blue dots indicated the upregulated genes and purple dots indicated the downregulated genes in organoid*^healthy liver^* between passage 3 and passage 7. **(C)** Volcano plot of organoid DEGs. Blue dots indicated the upregulated genes and purple dots indicated the downregulated genes in organoid*^PSC liver^* between passage 3 and passage 7.


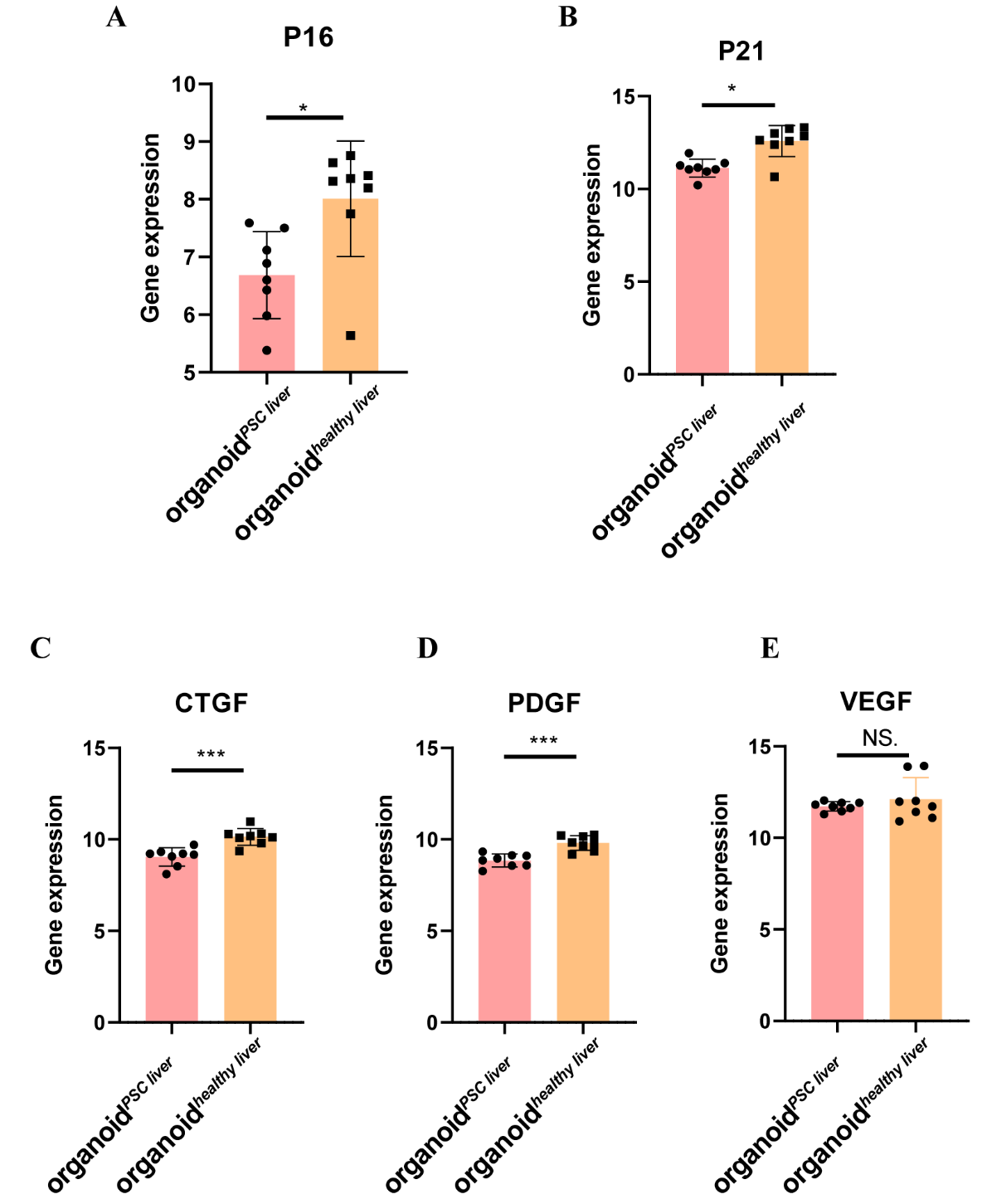


**Figure S7.** **Transcriptome analysis revealed the cholangiocytes senescence- related, profibrotic cytokines-related and angiogenesis in organoid*^PSC liver^*.** Histogram of cholangiocytes senescence-related **(A)** P16, **(B)** P21 gene expression between organoid*^PSC liver^* and organoid*^healty control^*. Histogram of cholangiocytes profibrotic cytokines-related **(C)** CTGF, **(D)** PDGF, and angiogenesis **(E)** VEGF gene expression. (n=8). no significant (ns.) > 0.05. *P < 0.05.


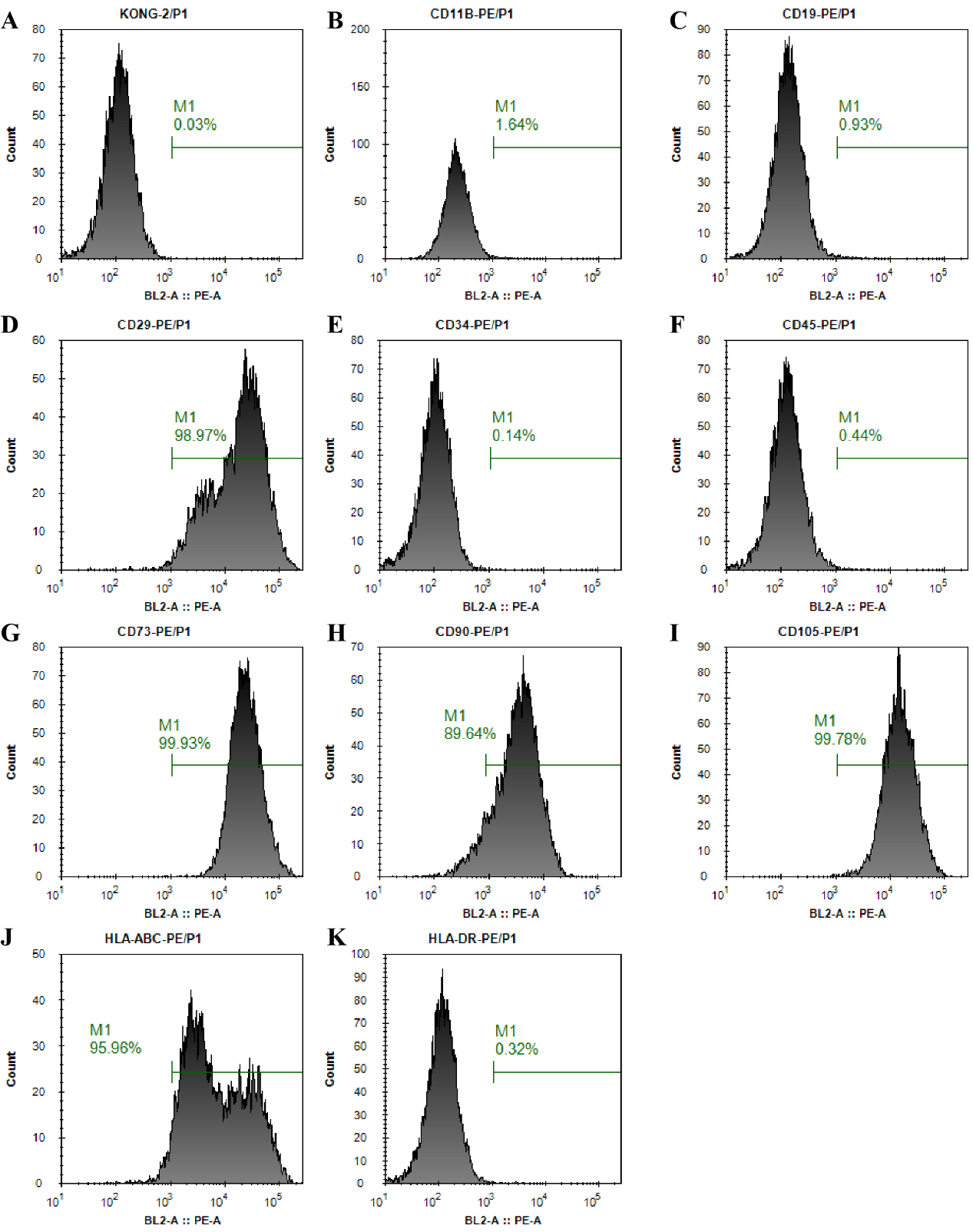


**Figure S8.** **Surface markers of hP-MSCs determined by flow cytometry.**

Surface markers of hP-MSCs determined by flow cytometry. **(A)** Control. **(B)** Cluster of Differentiation (CD)11B. **(C)** CD19. **(D)** CD29. **(E)** CD34. **(F)** CD45. **(G)** CD73. **(H)** CD90 **(I)** CD105. **(J)** HLA-ABC. **(K)** HLA-DR.


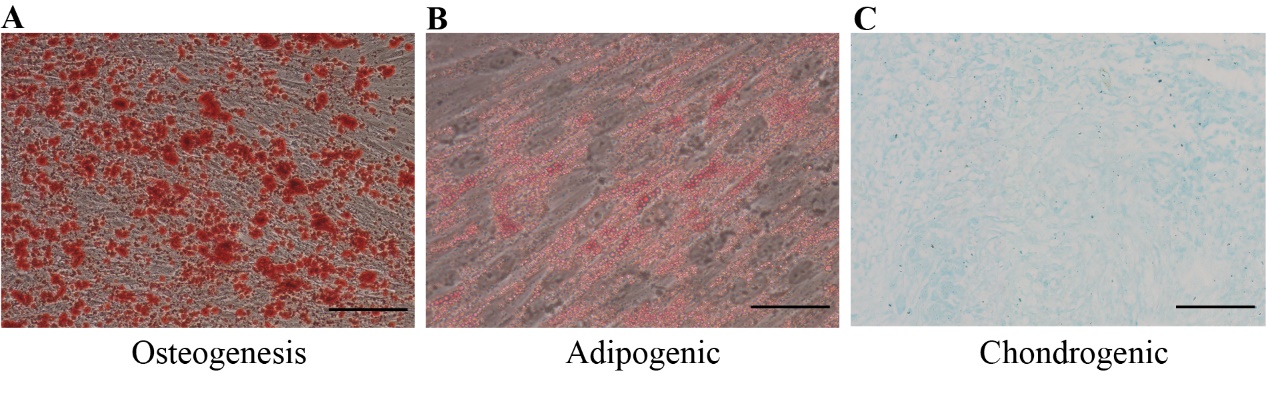


**Figure S9. Detection of hP-MSC multi-differential potential in *vitro*.**

**(A)** Alizarin red staining of osteogenesis differentiation. **(B)** Oil Red O staining of adipogenic differentiation. **(C)** Alcian blue staining of chondrogenic differentiation. Scale bar: 50 μm.


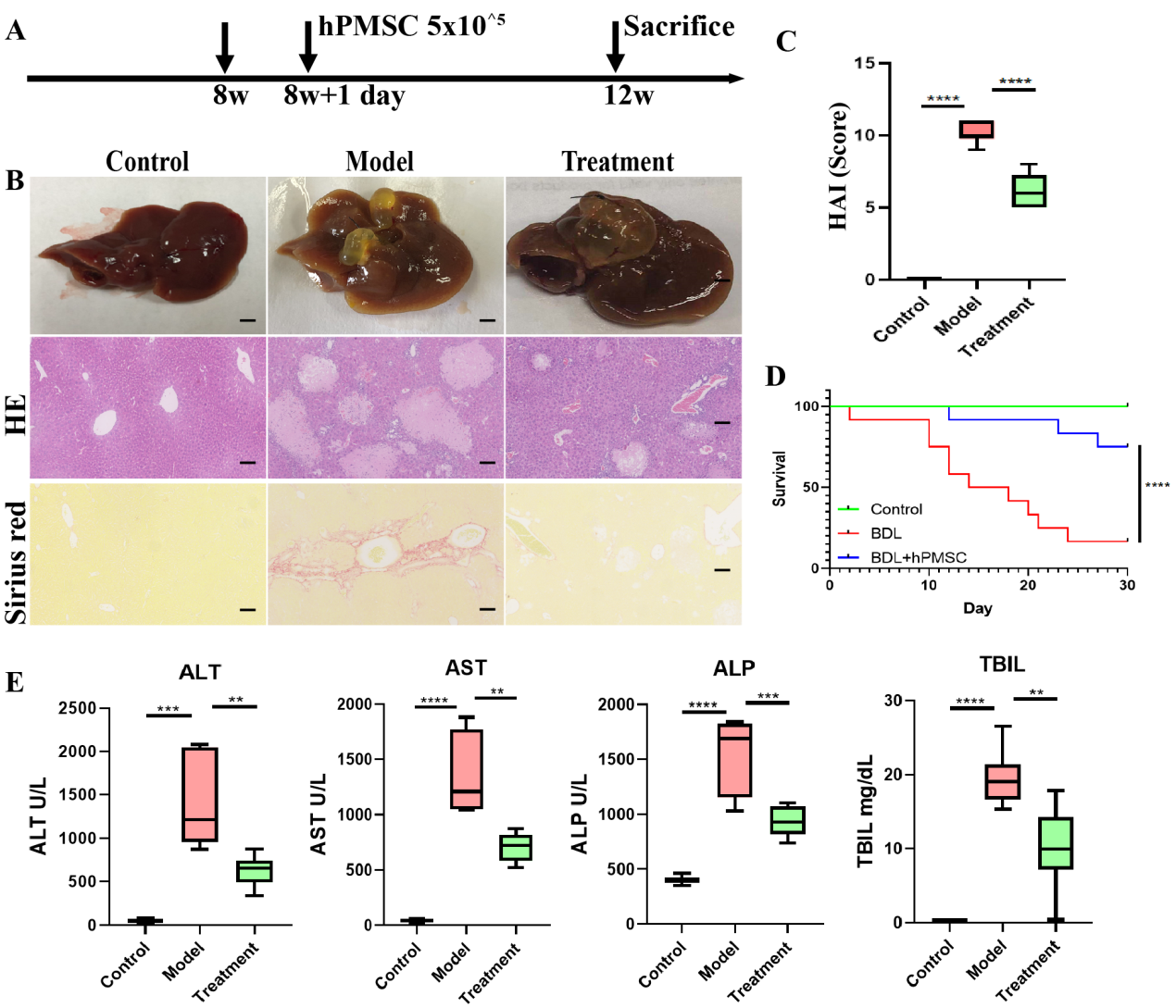


**Figure S10. hP-MSC treatment ameliorated the pathological process of sclerosing cholangitis BDL mouse models.**

**(A)** Schematic representation of the methodology used for treatment of Bile Duct Ligation (BDL) mice with hP-MSCs. C57Bl/6 mice (control group), BDL mice (model group), and hP-MSC-treated BDL mice (treatment group) (n = 30 for each group). **(B)** Analysis of hematoxylin and eosin-stained sections showed that hP-MSC treatment improved the inflammatory infiltration and cell necrosis in BDL mice. Sirius red staining showed that MSC treatment ameliorated liver fibrosis and collagen proliferation in mice. Scale bar: 100 μm. **(C)** Evaluation of therapeutic efficacy in DDC mice by HAI score. (n = 8 for each group). **(D)** Survival rates in the Control, BDL, BDL + hP-MSCs, after 30 days (*n* = 8 for each group). **(E)** Serum ALT, AST, ALP, and total bilirubin levels (n = 8 for each group). **P* < 0.05, ***P* < 0.01, ****P* < 0.001, *****P* < 0.0001.


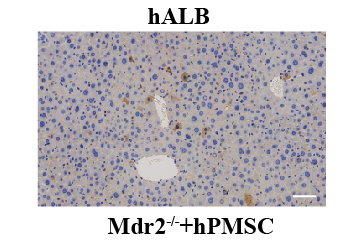


**Figure S11. hP-MSC treatment by tail vein injection in Mdr2^−/−^ mice**.

Mdr2^−/−^ mice were injected intravenously with hP-MSCs. hP-MSCs were observed to differentiate into hepatocytes at week 4 in Mdr2^−/−^ mice.


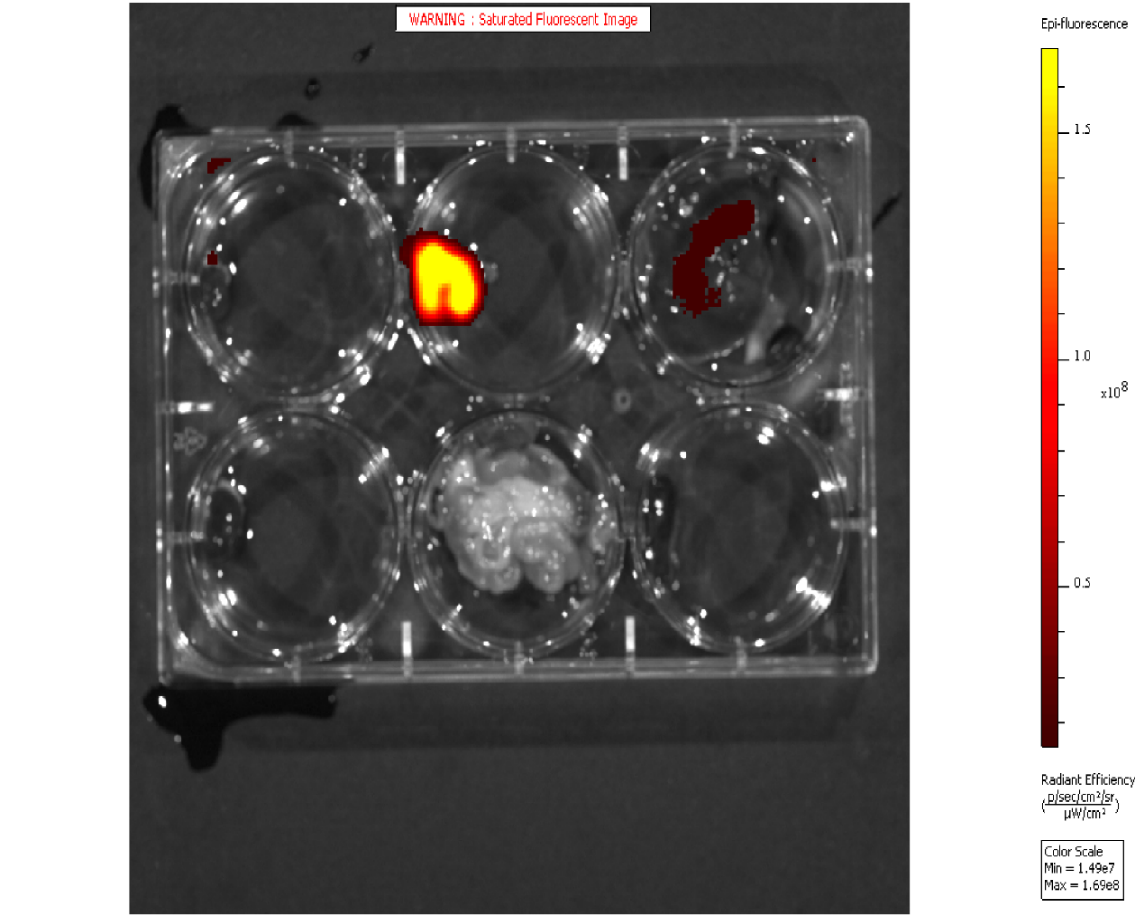


**Figure S12. hP-MSC treatment by tail vein injection in Mdr2^−/−^ mice**.

Mdr2^−/−^ mice were injected intravenously with hP-MSCs labeled with the fluorescent dye 1,1′-dioctadecyl-3,3,3′,3′-tetramethylindotricarbo-cyanine-iodide (DiR) for long-term follow-up.Fluorescence imaging of the lungs, heart, kidney, liver, and spleen of recipient mice injected with labeled hP-MSCs. DiR-labeled cells were mainly distributed in the lungs and liver, while no labeled cells were found in the heart, spleen, and intestine in Day 7.


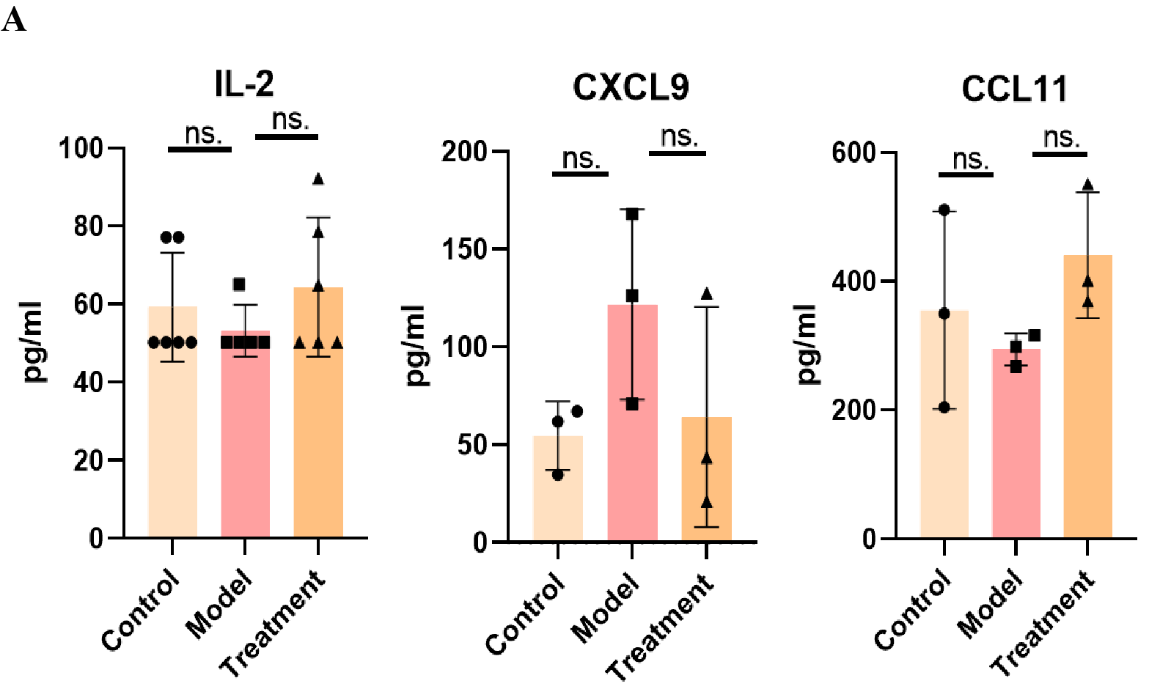


**Figure S13. hP-MSC treatment ameliorated the pathological process of Mdr2^−/−^ mice.**

Mdr2^+/+^ mice (control group), Mdr2^−/−^ mice (model group), and hP-MSC-treated Mdr2^−/−^ mice (treatment group). Detection of inflammatory factors (IL-2) and chemokines (CXCL9 and CCL11) among control, model and treatment groups of Mdr2^-/-^ mice. (n = 3-6 for each group). no significant (ns.) > 0.05.


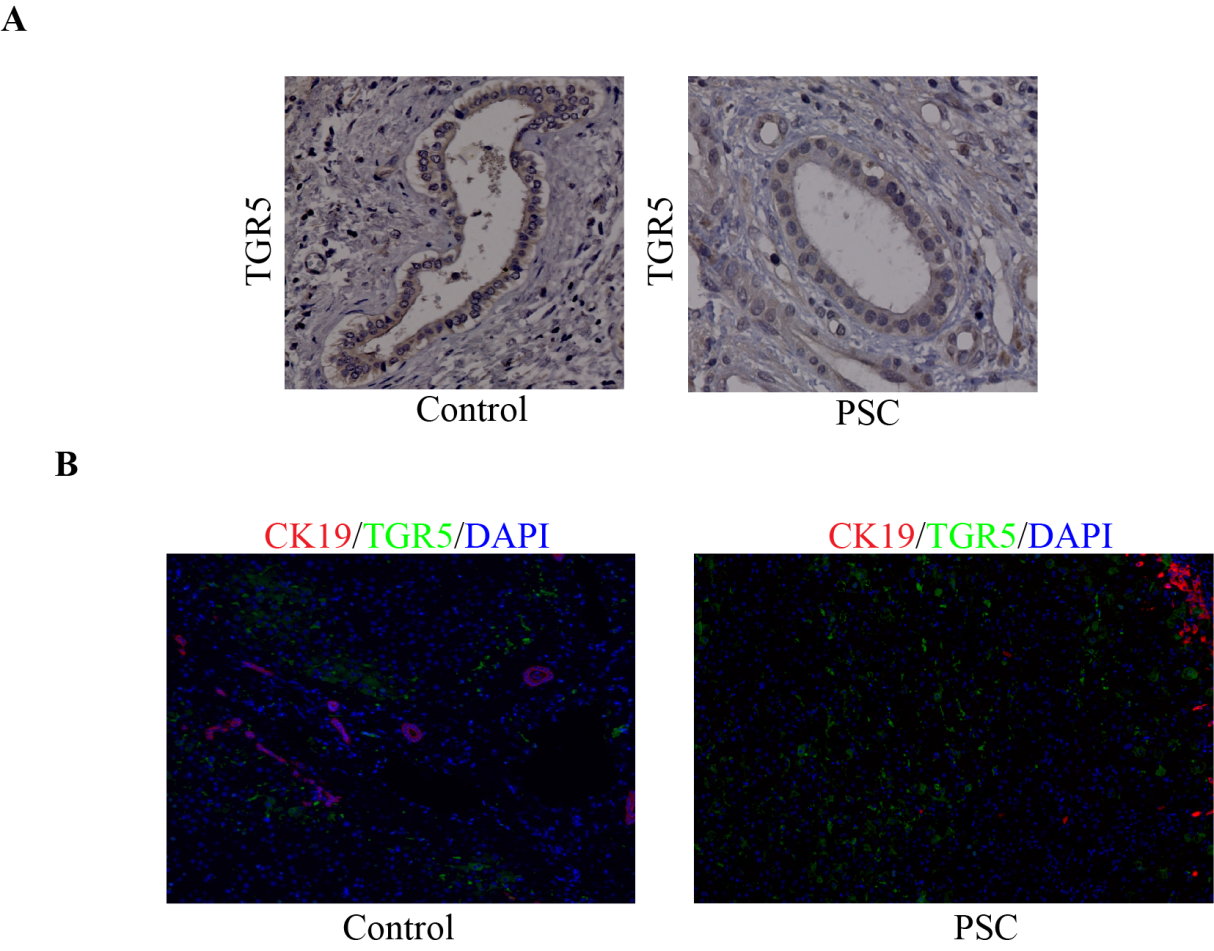


**Figure S14. Expression of TGR5 in liver tissues and organoids.**

**(A)** Immunohistochemical staining of TGR5 expression in liver tissue of PSC patients and healthy controls. **(B)** TSA staining of macrophages in liver tissue from PSC patients and healthy controls.


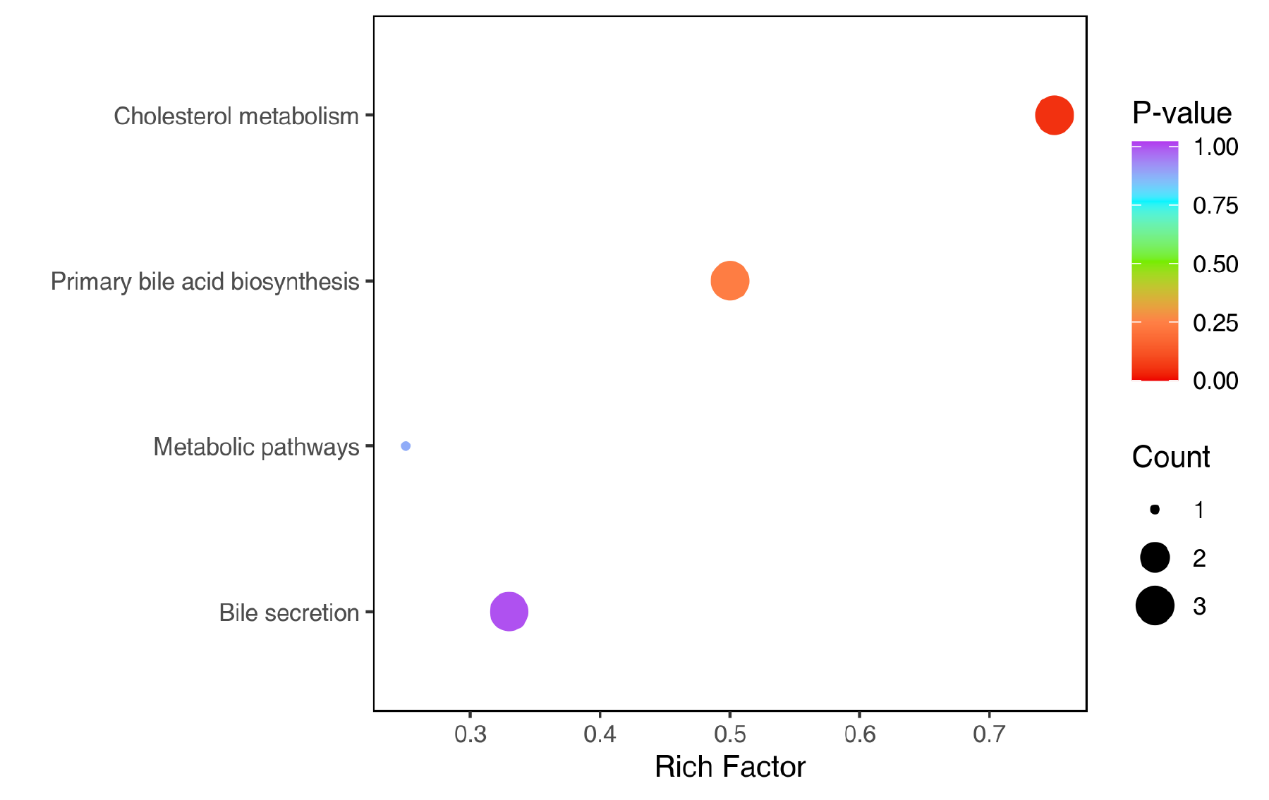


**Figure S15. Enrichment of differential metabolites of bile acids.**

Mdr2^+/+^ mice (control group), Mdr2^−/−^ mice (model group), and hP-MSC-treated Mdr2^−/−^ mice (treatment group) were analyzed by Liquid Chromatography/Mass Spectrometry KEGG analysis of differential metabolites of bile acids.


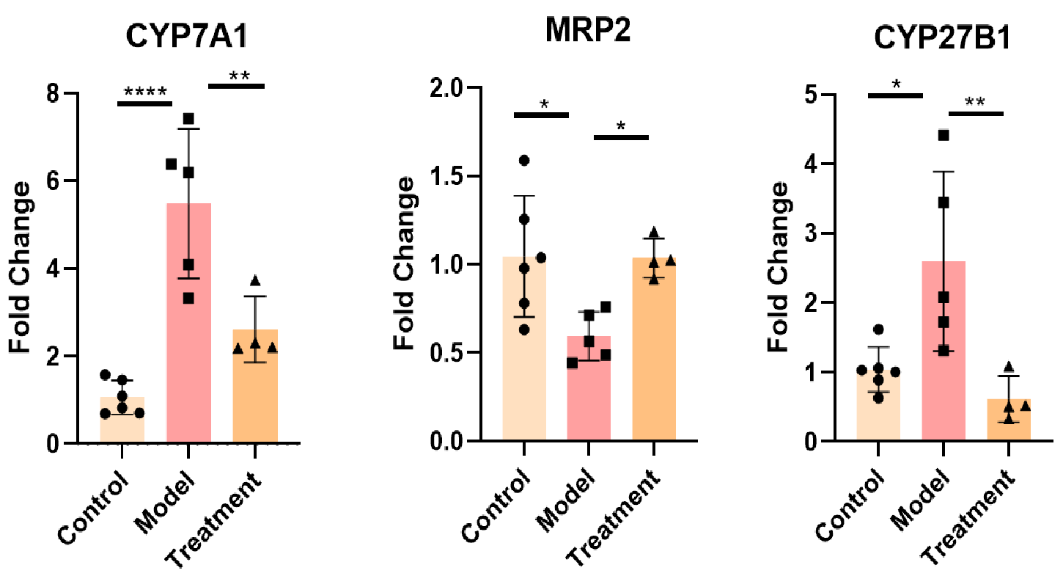


**Figure S16. hP-MSC treatment ameliorated bile acid metabolism in Mdr2^−/−^ mice.**

Mdr2^+/+^ mice (control group), Mdr2^−/−^ mice (model group), and hP-MSC-treated Mdr2^−/−^ mice (treatment group) were analyzed by Liquid Chromatography/Mass Spectrometry. Treatment with hP-MSCs ameliorated the changes in expression of genes related to bile acid metabolism (CYP7A1, CYP27B1, and MRP2) in Mdr2^−/−^ mice (n = 4-6 for each group). **P* < 0.05, ***P* < 0.01, *****P* < 0.0001.


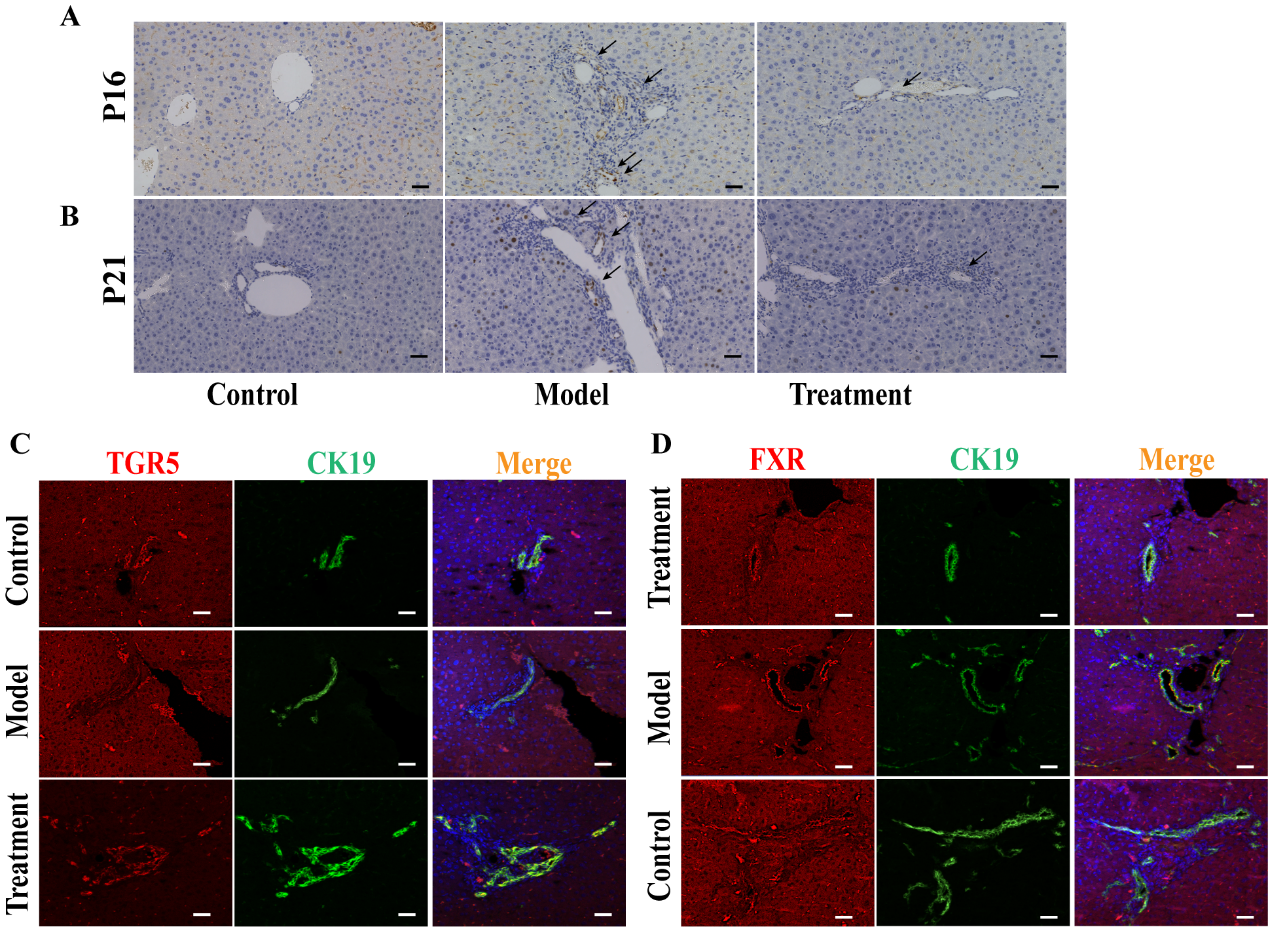


**Figure S17. Efficacy of hP-MSC treatment in Mdr2^−/−^ mouse model of sclerosing cholangitis.**

**(A)** Immunohistochemistry staining of P16 in cholangiocytes of Mdr2^−/−^ and Mdr2^+/+^ mice. Scale bar: 100 μm. **(B)** Immunohistochemistry staining of P21 in cholangiocytes of Mdr2^−/−^ and Mdr2^+/+^ mice. Scale bar: 100 μm. **(C)** Fluorescence staining of TGR5 and CK 19 in cholangiocytes of Mdr2^−/−^ and Mdr2^+/+^ mice. Scale bar: 100 μm. **(D)** Fluorescence staining of FXR and CK 19 in cholangiocytes of Mdr2^−/−^ and Mdr2^+/+^ mice. Scale bar: 100 μm.


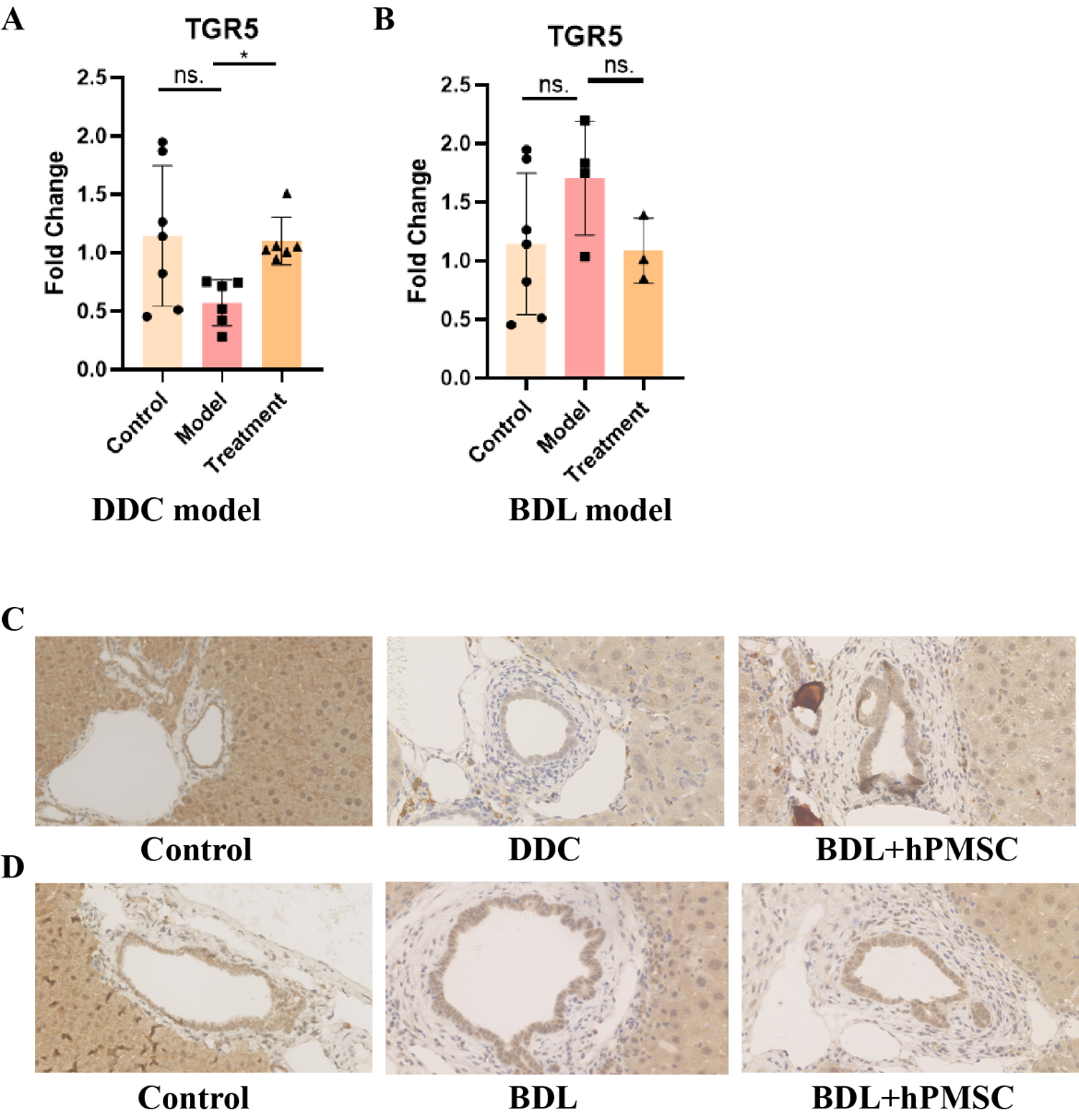


**Figure S18. Expression of TGR5 in cholangiocytes in hP-MSC-treated DDC and BDL models.**

**(A)** TGR5 mRNA levels in the hP-MSC-treated DDC mouse model. C57Bl/6 mice (control group), 0.1% 3,5-diethoxycarbonyl-1,4-dihydrocollidine (DDC) fed mice (model group), and hP-MSC-treated DDC mice group) (n = 6-7 for each group). **(B)** TGR5 mRNA levels in the hP-MSC-treated BDL mouse model. C57Bl/6 mice (control group), BDL mice (model group), and hP-MSC-treated BDL mice (treatment group) (n = 6-7 for each group). **(C)** Immunohistochemical analysis of TGR5 protein expression in the hP-MSC-treated DDC mouse model. **(D)** Immunohistochemical analysis of TGR5 protein expression in the hP-MSC-treated BDL mouse model. **P* < 0.05. no significant (ns.) > 0.05.


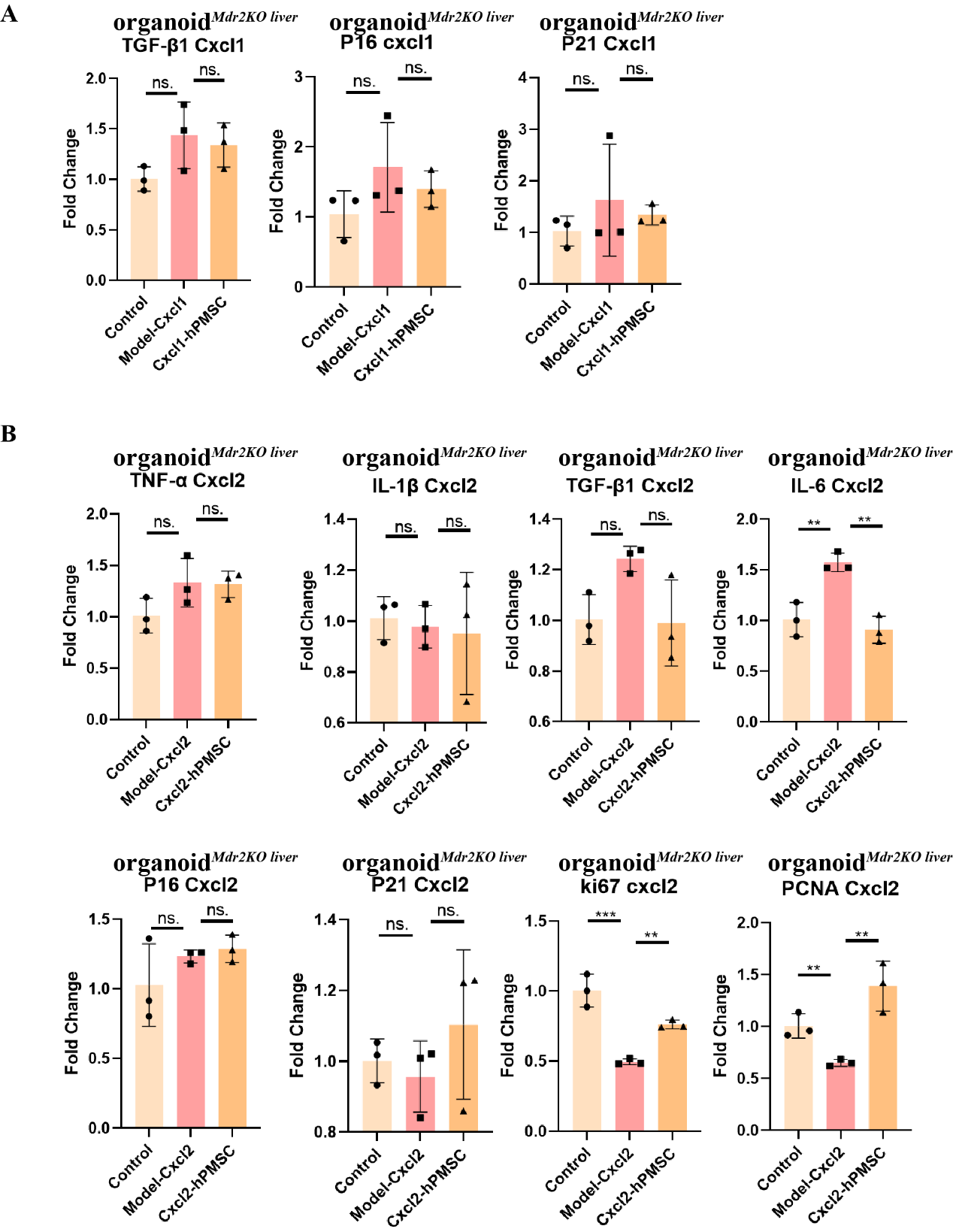


**Figure S19. Changes in mRNA expression in organoid*^Mdr2KO liver^* co-cultured with hP-MSCs *in vitro*.**

We added CXCL1, CXCL2 separately to organoid*^Mdr2KO liver^* culture medium at 24 h, followed by co-culture with hP-MSCs for a further 24 h. **(A)** Histogram comparing expression of genes related to inflammation (TGF-β1) and senescence (p16 and p21) between organoid*^Mdr2KO liver^*, organoid*^Mdr2KO liver^*-CXCL1, and hP-MSC-organoid*^Mdr2KO liver^*-CXCL1 groups (n = 3 for each group). **(C)** Histogram comparing expression of genes related to inflammation (TNF-α, IL-1β, TGF-β1, and IL-6), proliferation (Ki-67 and PCNA), and senescence (p16 and p21) between organoid*^Mdr2KO liver^*, organoid*^Mdr2KO liver^*-CXCL2, and hP-MSC-organoid*^Mdr2KO liver^*-CXCL2 groups (n = 3 for each group). **P* < 0.05, ***P* < 0.01. no significant (ns.) > 0.05.
